# Examining tools for assessing the impact of chronic pain on emotional functioning in children and young people with cerebral palsy: stakeholder preference and recommendations for modification

**DOI:** 10.1007/s11136-024-03693-1

**Published:** 2024-05-25

**Authors:** Meredith Grace Smith, Rachel J. Gibson, Remo N. Russo, Sophie Karanicolas, Adrienne R. Harvey

**Affiliations:** 1 School of Allied Health Science and Practice, University of Adelaide, North Terrace, Adelaide, SA 5005 Australia; 2 College of Medicine and Public Health, Flinders University, Adelaide, Australia; 3 Paediatric Rehabilitation Department, Women’s and Children’s Hospital, Adelaide, Australia; 4 School of Dentistry, University of Adelaide, Adelaide, Australia; 5 Neurodisability and Rehabilitation, Murdoch Children’s Research Institute, Melbourne, Australia; 6 Medicine, Dentistry and Health Sciences, University of Melbourne, Melbourne, Australia

**Keywords:** Measurement, Chronic pain, Cerebral palsy, Patient-reported outcome measures

## Abstract

**Purpose:**

To firstly identify tools for assessing the impact of chronic pain on emotional functioning in children and young people with cerebral palsy (CP), and secondly identify suggestions to improve their relevance, comprehensiveness, comprehensibility and feasibility for the CP population. Improving assessment of the impact of pain on emotional functioning can enhance quality of life by improving access to interventions for pain-related physical disability, anxiety and depression.

**Methods:**

Ethics approval was granted through the Women’s and Children’s Health Network Human Research Ethics Committee (2022/HRE00154). A mixed methods study with people with lived experience and clinicians, and guided by the Consensus-based Standards for Measurement Instruments (COSMIN), was undertaken. An online survey identified the highest rated tools for validation and/or modification for young people with CP and chronic pain. Focus groups and interviews investigated content validity and feasibility of the tools identified as highest rated.

**Results:**

The Fear of Pain Questionnaire for Children-SF (FOPQ-C-SF) and Modified Brief Pain Inventory (mBPI) were the highest rated for pain coping and multidimensional assessment (respectively) from the online survey (*n* = 61) of eight tools presented. Focus group and interview data (*n* = 30), including 58 unique modification suggestions, were coded to six categories: accessibility, comprehensibility, feasibility, relevance, presentation and comprehensiveness.

**Conclusion:**

Potential modifications have been identified to improve the appropriateness and feasibility of the FOPQ-C-SF and mBPI for children and young people with CP. Future research should implement and test these modifications, prioritising the involvement of people with lived experience to ensure their needs are met alongside clinicians.

**Supplementary Information:**

The online version contains supplementary material available at 10.1007/s11136-024-03693-1.

## Abstract (plain language summary)

Up to 75% of children and young people with cerebral palsy report chronic pain, which is much higher than those without cerebral palsy. Assessing how pain impacts emotional functioning, and how each individual copes with pain, is of particular importance due to known links between emotional functioning and long term pain outcomes. Reliable assessment of how pain impacts emotional functioning may also help to identify those who would benefit from psychological treatments. Although pain questionnaires are available, many are not suitable for children and young people with cerebral palsy with different communication, cognitive and movement abilities. This study had two aims: (1) to work out which of the currently available tools that assess how pain impacts emotional functioning are considered best for people with cerebral palsy, and (2) to identify potential modifications to these tools. The two most relevant and easy to understand questionnaires selected for modification were the Fear of Pain Questionnaire for Children and the modified Brief Pain Inventory. A number of modifications were identified, including improving how relevant the questions were to people with cerebral palsy, improving accessibility for people with complex communication needs or cognitive impairment and improving how easy to understand the questions and answer options are. These modifications can now be implemented to make it easier for people with cerebral palsy to use the pain assessments. They should then be tested in people with cerebral palsy with different communication, cognitive and movement abilities.

**Supplementary Information:**

The online version contains supplementary material available at 10.1007/s11136-024-03693-1.

## Introduction

Cerebral palsy (CP) is the leading cause of childhood disability, with a global prevalence of 1.6/1000 live births [1]. Young people with CP experience chronic pain more commonly than other populations, with rates between 14% and 76% of the population [2]. Despite this, chronic pain in CP remains poorly understood and managed [3].

Inadequate chronic pain assessment in CP may stem from underutilisation of assessment tools [4]. Harvey et al. (2021) established consensus on twelve core domains for chronic pain assessment in CP [5], however few validated tools exist [6]. Furthermore, available tools lack suitability across the varying cognitive, communication and motor abilities in CP [7, 8]. Recent reviews highlight a particular need for valid tools assessing impact of pain on emotional functioning, particularly pain coping [6, 8]. Pain coping includes three key psychological factors (pain anxiety, pain catastrophising and fear of pain) which influence pain-related physical disability, anxiety and depression, all with significant impact on a person’s quality of life [9, 10]. These factors are also targets for psychological interventions, influencing outcomes of the domain, ‘impact of pain on emotional wellbeing’ [11]. Multidimensional tools assessing impact of pain on emotional functioning may assist in identifying individuals who would benefit from further assessment of pain coping, and subsequently improve access to currently underutilised pain coping interventions [12, 13].

Content validity is an essential measurement property of any tool; ensuring a tool measures what it claims to measure. This includes relevance to the intended population and construct, comprehensiveness ensuring no key aspects of the construct are missing, and comprehensibility to ensure items are understood as intended [14]. Feasibility is also key for integration into clinical practice [14]. Modifying pain assessment tools for children and young people with CP may improve content validity, feasibility, opportunity for self-report of the individual pain experience, and increase access to best practise pain interventions, thus improving quality of life [15].

The primary objective of this mixed methods study, comprising of an online survey and a qualitative descriptive study, was to determine which of the currently available tools assessing impact of pain on emotional functioning would be most suitable to modify and/or validate in young people with CP. Secondary objectives were to identify modifications required for improving appropriateness and feasibility of these tools, and to investigate content validity and feasibility of these tools in young people with CP.

## Methods

### Study design

This study was approved by the Women’s and Children’s Health Network Human Research Ethics Committee (2022/HRE00154) and was guided by COnsensus-based Standards for Measurement Instruments (COSMIN) recommendations for investigating content validity. Further, this study was informed by an advisory group of people with lived experience, including two young adults with CP and a parent of a child with CP.

Between January and April 2023, a two-stage mixed methods study was undertaken. In Stage 1, an online survey identified suitable tools for assessing the impact of pain on emotional functioning with potential for modification and/or validation. In stage 2, a qualitative descriptive study explored content validity, clinical feasibility and tool modification options. The consolidated criteria for reporting qualitative research (COREQ) were followed [16].

### Participants and recruitment

Participants in both stages were clinicians (physiotherapists, occupational therapists, psychologists, clinician researchers, speech pathologists, paediatricians, orthopaedic surgeons), individuals with CP (> 8 years of age, the minimum age the tools had been developed for) and parents of children with CP (Table 1). All participants (stage 1 and 2) were recruited by online advertising and flyer distribution through the Australasian Academy for Cerebral Palsy and Developmental Medicine (AusACPDM), Novita, the Women’s and Children’s Hospital and the South Australian CP Register. Purposeful sampling was employed to facilitate inclusion of varied motor ability, communication ability and age in the qualitative component [17].

### Sample size

The expected maximum sample size for the survey was 60 clinicians and 30 people with lived experience, based on AusACPDM membership. For the qualitative study, the target sample size was 15–20 individuals, including at least 7 individuals with CP. COSMIN consider testing content validity in a qualitative study of seven or more participants from the population of interest as ‘very good’ [18].

**Table 1:** Study eligibility criteria

Study component	Inclusion criteria	Exclusion criteria
Online survey	Individuals with a diagnosis of CP > 8 years of age, any severity (GMFCS levels I-IV)	
	Parents or caregivers of children with CP (child age = 2–30 years)	
	Medical, nursing or allied health clinicians or clinician researchers with > 5 years’ experience assessing and treating CP	Clinicians with < 5 years’ experience assessing and treating CP
	English speaking and reading ability	
Qualitative descriptive study	Individuals with a diagnosis of CP > 8 years of age, any severity (GMFCS levels I-IV)	
	Parents or caregivers of children with CP (child age = 2–30 years)	
	Medical, nursing or allied health clinicians or clinician researchers with > 5 years’ experience assessing and treating CP	Clinicians with < 5 years’ experience assessing and treating CP
	English speaking ability	Unable to communicate verbally or with a communication device in a focus group or interview setting

## Procedure

### Stage 1: online survey

Survey data were collected and managed using REDCap electronic data capture tool, hosted at Murdoch Children’s Research Institute [19, 20]. The survey was open for four weeks.

#### Survey instrument

**Fig. 1:**
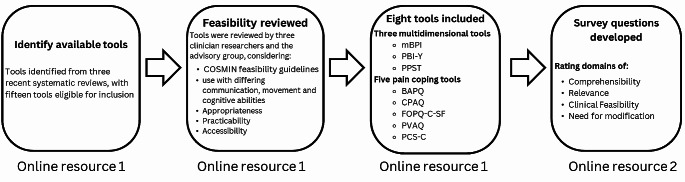
Development of the survey instrument (further detail provided in Online Resource 1 & 2) **Abbreviations:bapq** = Bath Adolescent Pain Questionnaire, **cpaq** = Chronic Pain Acceptance Questionnaire, **fopq** = Fear of Pain Questionnaire for Children Short Form, **mbpi** = Modified Brief Pain Inventory, **pbi** = Pain Burden Inventory, **ppst** = Pediatric Pain Screening Tool, pcs = Pain Catastrophizing Scale for Children, **pvaq** = Pain Vigilance and Awareness Questionnaire

The development of the survey included four main stages (Fig. 1, Online Resource 1 & 2). Available pain tools measuring impact of pain on emotional functioning were identified from three recent systematic reviews [6–8]. Pain coping tools were defined as those that assessed pain coping, including pain anxiety, pain catastrophising and fear of pain within a dedicated scale or subscale [9, 10]. Multidimensional tools were defined as assessing many pain domains in CP, with at least one item assessing impact of pain on emotional functioning, defined as “the extent to which pain hinders engagement with emotional functioning” [21]. As advisory group members recommended no more than eight tools be presented in the survey, tools were reviewed for feasibility [22, 23] and the following tools identified for inclusion; three multidimensional tools (Modified Brief Pain Inventory (mBPI) [24], Pain Burden Inventory-Youth (PBI) [25], Pediatric Pain Screening Tool (PPST) [26]) and five pain coping tools (Bath Adolescent Pain Questionnaire (BAPQ) [27], Chronic Pain Acceptance Questionnaire (CPAQ) [28], Fear of Pain Questionnaire for Children-Short Form (FOPQ-C-SF) [29], Pain Vigilance and Awareness Questionnaire (PVAQ) [30] and Pain Catastrophizing Scale for Children (PCS-C) [31]).

#### Data analysis

Stage 1 determined the highest-rated tool for (1) pain coping and (2) multidimensional assessment, for further exploration in stage 2. Data were analysed by the percentage of ‘strongly agree’ and ‘agree’ responses for relevance, comprehensibility, clinical feasibility and need for modification of each tool (Online Resource 3). Equal weighting was given across clinicians/researchers and lived experience participants, except for comprehensibility which was rated only by people with lived experience in line with COSMIN [18].

### Stage 2: qualitative study

A qualitative descriptive study was undertaken to summarise the relevance, comprehensiveness, comprehensibility and feasibility of key tools assessing impact of pain on emotional functioning in children and young people with CP and chronic pain [32, 33]. Stakeholder recommendations to improve routine clinical use of chronic pain assessment tools were also identified.

#### Data collection methods

Adult participants were offered the choice of a focus group or individual interview, with separate groups for people with lived experience and clinicians. All children were interviewed to ensure they were able to express their views clearly [34]. Focus groups lasted 60 min and were conducted by a single interviewer (SK). Semi-structured interviews lasted 30–60 min and were conducted by a single interviewer (MGS). All focus groups and interviews were undertaken online using video conferencing software, digitally recorded and transcribed verbatim. Interviews with children (< 18 years) were conducted with parents present, based on child/parent preference.

Participants were provided with a copy of the tools one week prior. Participants were advised they were able to cease or pause the interview at any time. A semi-structured interview guide was developed [34], and informed by both COSMIN requisites for content validity and stage 1 open-ended survey responses (Table 2, Online Resource 4) [18, 35]. The interview guide underwent rigorous testing prior to use: internal testing within the author team, and critique and field testing with the advisory group [34].

**Table 2:** Key topics and prompts in the semi structured interview guide

Topic	Prompts	Participant group
Relevance	e.g. “Which questions are relevant to pain for people with cerebral palsy?”	All
Comprehensibility	e.g. “How could we make the questions easier to understand?”	All
Comprehensiveness	e.g. “Is there anything important or unique to people with cerebral palsy that is missing from this questionnaire?”	All
Clinical feasibility	e.g. “What suggestions would you make to improve the layout of this tool?”	All
Clinical feasibility	e.g. “How would you feel if you were asked to complete this tool before or during an appointment with your health professional?”	Parents/people with CP
Clinical feasibility	e.g. “How would you incorporate a tool like this into your clinical practice?”	Clinicians

#### Data analysis

Inductive content analysis was used to determine practical and concrete suggestions to inform practice [33, 36]. NViVO 12.0plus software (QSR International, Melbourne, Australia) was used to facilitate analysis. Briefly, an initial period of familiarisation was undertaken by two authors (MGS, ARH), whereby recordings were listened to, transcripts read and exploratory notes recorded. Coding was completed in two rounds, independently, by two authors (MGS, ARH). These authors met initially after completing two transcripts, then regularly to compare categories applied and to ensure consistent terminology. In the first round, transcripts were coded to identify big picture meaning units and develop a preliminary big picture coding schema [36]. The second round coding used a ‘line by line’ process to produce subcategories, resulting in a refined coding schema (Online Resource 5), which was reviewed by an external qualitative advisor (AC). Each suggested modification was identified and mapped to the big picture categories.

#### Researcher characteristics and reflexivity

Inductive content analysis recognises the researcher’s perspectives and prior experiences may influence data analysis [36]. MGS is a female PhD student and physiotherapist, and has training in qualitative research. SK is an experienced female qualitative researcher and education specialist. ARH, RR and RJG supervised the research. ARH is an experienced disability researcher, physiotherapist and also has training in qualitative research. RR is a paediatric rehabilitation medical specialist and researcher, with > 30 years’ experience working with children with CP. RJG is an experienced researcher with > 20 years’ experience in both quantitative and clinical studies.

#### Trustworthiness

Credibility was demonstrated by establishing rapport with patients prior to commencing interviews, with some participants already known to MGS through clinical relationships. Transcripts and quotes were reviewed by participants. Using focus groups alongside interviews reduced the role of the researcher, improving authenticity. Including participant demographics to demonstrate the breadth of age, communication ability, cognitive ability and motor ability represented in the study, establishes transferability. Direct quotes were included to ensure confirmability of the findings. Qualitative data, including the final coding schema, were triangulated with online survey open-ended responses and advisory group.

## Results

### Participant characteristics

Sixty-one participants completed the online survey (32 clinicians, 19 parents and 10 individuals with CP) and 30 participants undertook the qualitative descriptive study (12 clinicians, 9 parents and 9 young people with CP [12–39 years of age]) (Table 3). No participants withdrew from the study.

**Table 3:** Descriptive statistics of participants

	Online survey (*n* = 61) % (n)	Qualitative descriptive study (*n* = 30) % (n)
**Clinicians**	**52.5% (32)**	**40% (12)**
*Occupational Therapist*	*1.6% (1)*	*-*
*Orthopaedic Surgeon*	*3.2% (2)*	*-*
*Paediatrician*	*6.6% (4)*	*3.3% (1)*
*Physiotherapist*	*36.1% (22)*	*26.7% (8)*
*Psychologist*	*1.6% (1)*	*3.3% (1)*
*Speech Pathologist*	*1.6% (1)*	*3.3% (1)*
*Other (clinician researcher)*	*1.6% (1)*	*3.3% (1)*
**People with lived experience of CP**	**47.5% (29)**	**60% (18)**
*Young people with CP* *[Age range]*	*16.4% (10)^*	*30% (9)* *[12–39 years]*
*Parents of children with CP* *[age range of child]*	*31.1% (19)*	*30% (9)* *[4–25 years]*
Mobility Level (percentages based on the lived experience participants only)		
	*n* = 29	*n* = 18
*GMFCS I*	*13.8% (4)*	*5.6% (1)*
*GMFCS II*	*41.4% (12)*	*50% (9)*
*GMFCS III*	*17.2% (5)*	*11.1% (2)*
*GMFCS IV*	*6.9% (2)*	*11.1% (2)*
*GMFCS V*	*20.7% (6)*	*22.2% (4)*
Communication ability (percentages based on lived experience participants only)		
	*n* = 29	*n* = 18
*Able to report and describe pain without any additional assistance*	*75.9% (22)*	*72.2% (13)*
*Able to report and describe pain with the use of a communication device or other method*	*6.9% (2)*	*11.1% (2)*
*Unable to report and/or describe pain*	*17.2% (5)*	*16.7% (3)*
School type (percentages based on the child participants only *n* = 3)		
*Mainstream*		*66.7% (2)*
*Special unit*		*33.3% (1)*

^No children with CP (< 18 years) participated in the online survey, age data for > 18 years was not collected for the survey

### Stage 1: online survey

**Fig. 2:**
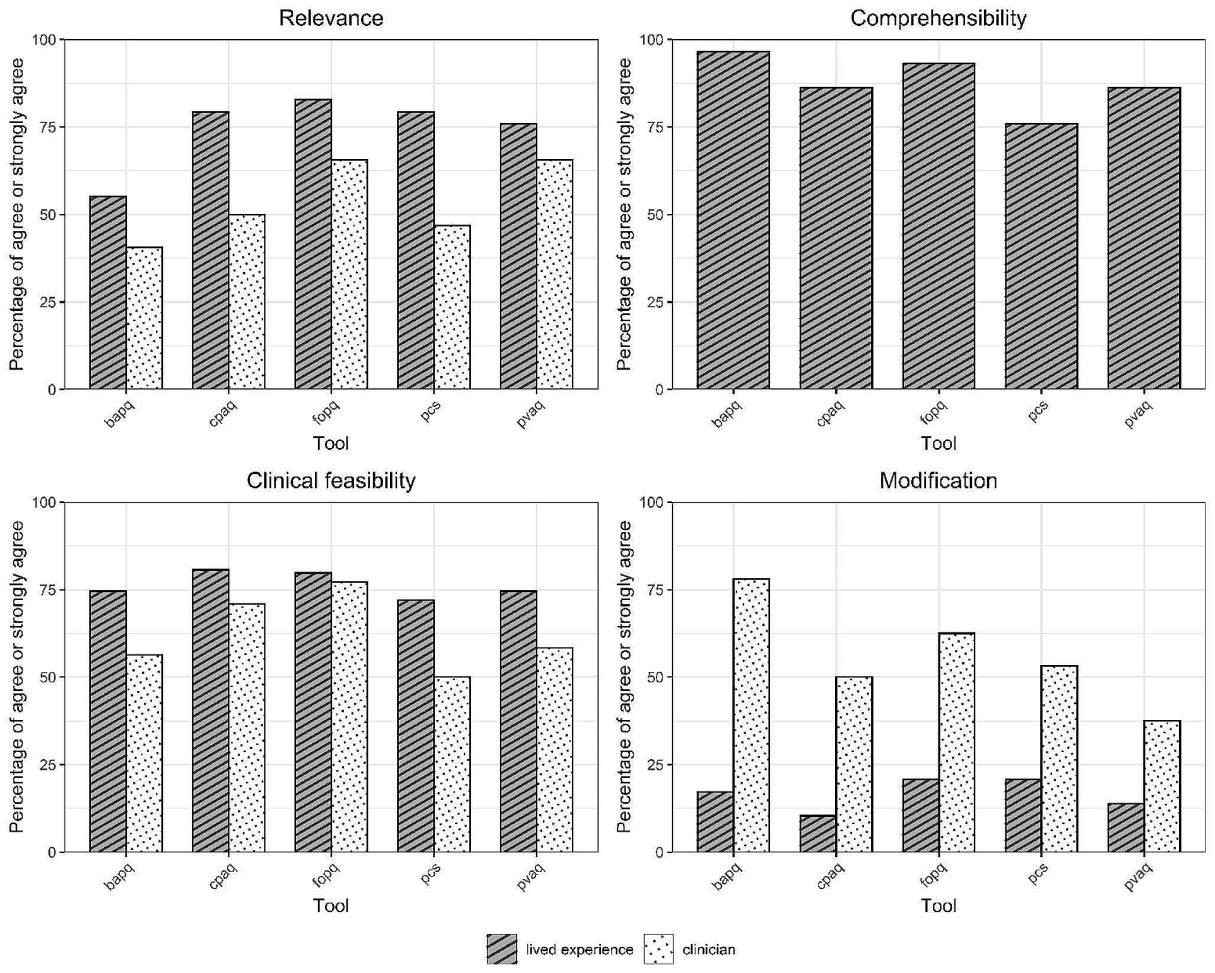
Ratings of relevance, comprehensibility, clinical feasibility and the need for modification of pain coping tools **Abbreviations: bapq** = Bath Adolescent Pain Questionnaire, **cpaq =** Chronic Pain Acceptance Questionnaire, **fopq =** Fear of Pain Questionnaire for Children Short Form, **pcs =** Pain Catastrophizing Scale for Children, **pvaq =** Pain Vigilance and Awareness Questionnaire

**Fig. 3:**
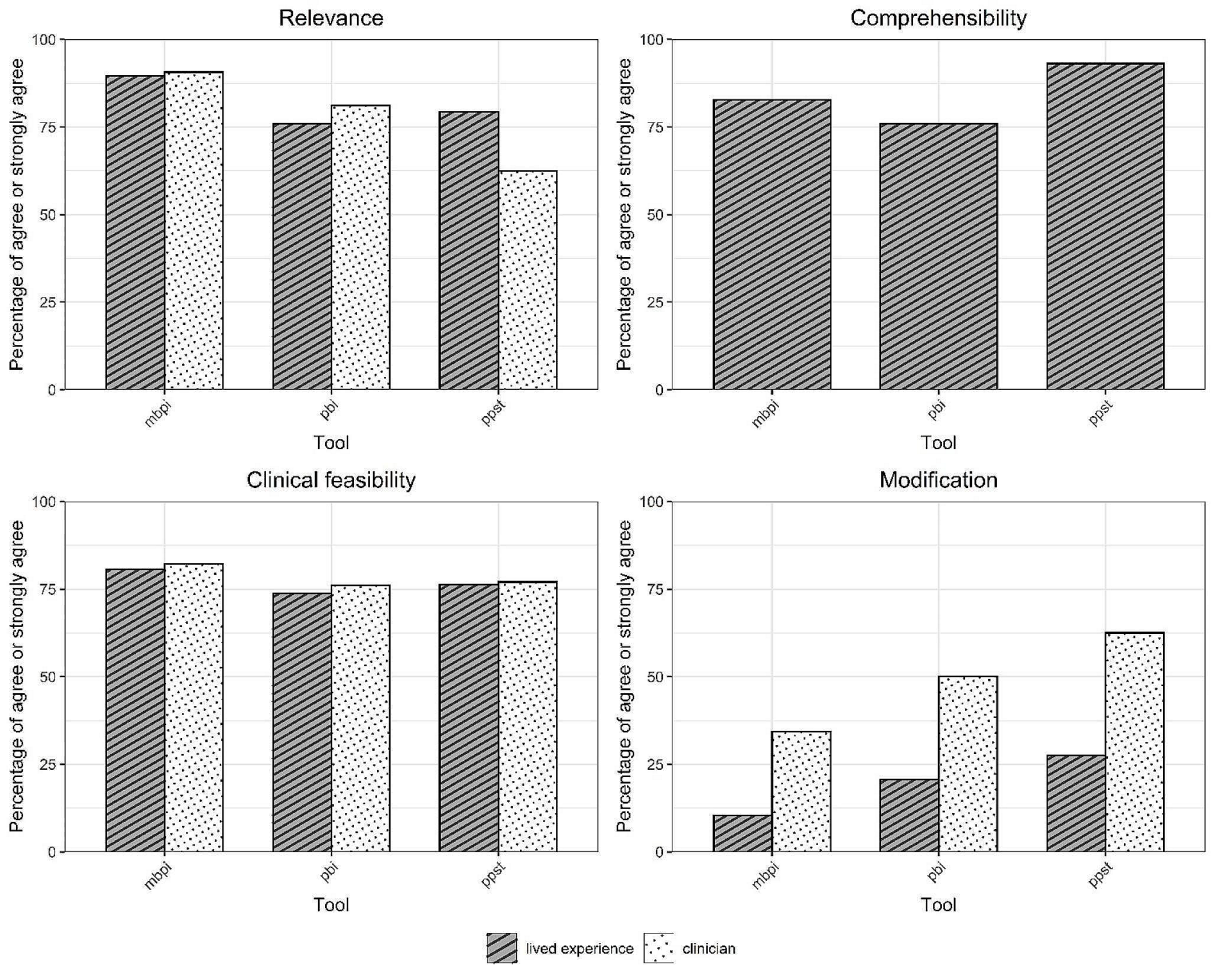
Ratings of relevance, comprehensibility, clinical feasibility and the need for modification of the multidimensional tools for chronic pain including assessment of ‘impact of pain on emotional functioning’ **Abbreviations: mbpi** = Modified Brief Pain Inventory, **pbi =** Pain Burden Inventory, **ppst =** Pediatric Pain Screening Tool

The FOPQ-C-SF was the highest rated pain coping tool for both relevance and clinical feasibility (Fig. 2, Online Resource 6) whereas the mBPI was the highest rated multidimensional tool for both relevance and clinical feasibility (Fig. 3). Both the FOPQ-C-SF and mBPI were second highest rated for comprehensibility (Figs. 2 and 3).

### Stage 2: qualitative study

**Fig. 4:**
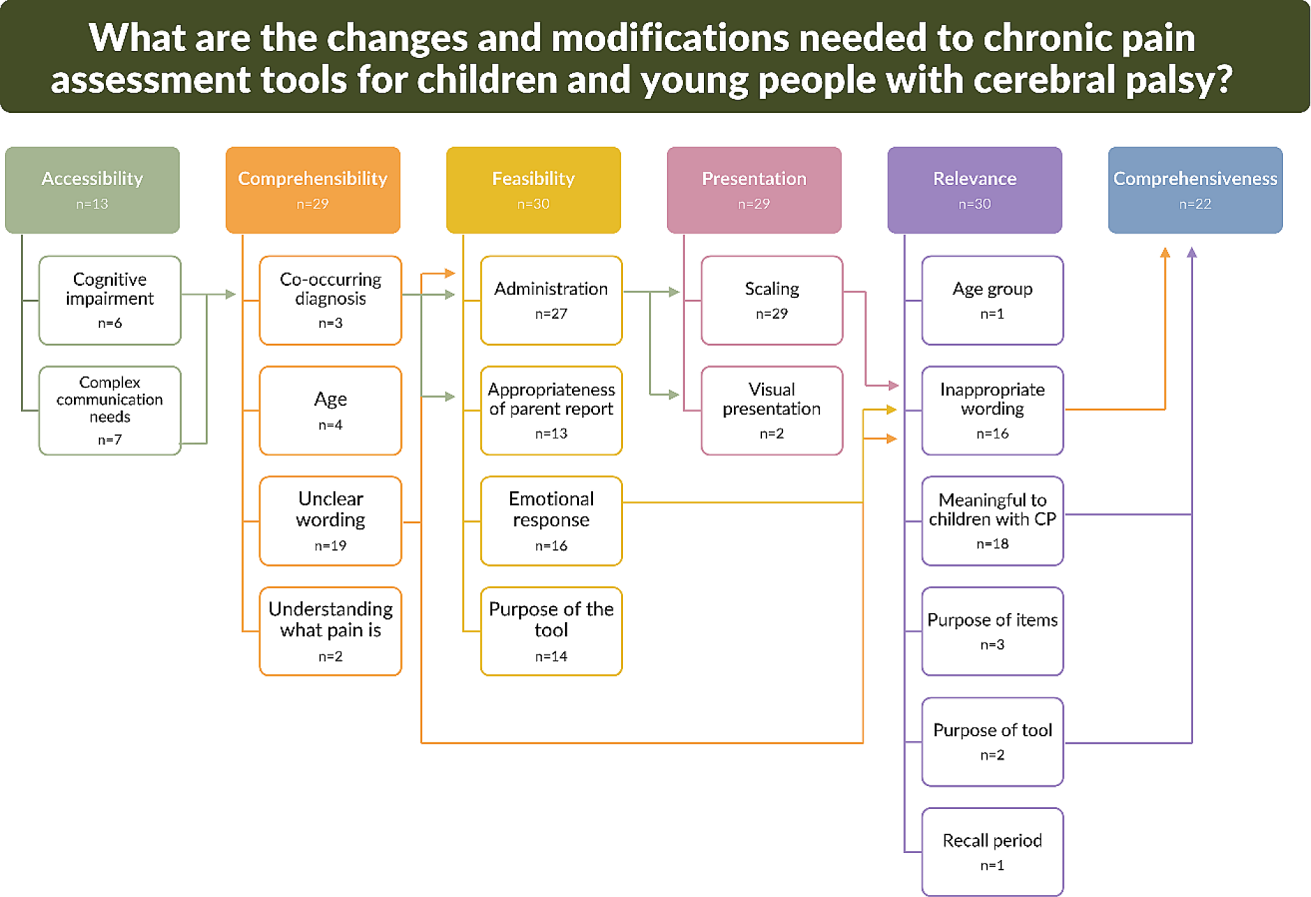
Categories from the qualitative descriptive study. Number of participants contributing data to each category is depicted by n = X

Five focus groups (three lived experience and two clinician groups) and eight interviews (two clinicians, one parent and five people with CP) were completed. Participant data were coded to six categories: accessibility, comprehensibility, feasibility, relevance, presentation and comprehensiveness (Fig. 4; Table 4). People with lived experience provided feedback primarily focused on relevance and comprehensibility, while clinicians provided feedback focused on presentation and comprehensibility. Fifty-eight unique modification suggestions were identified, 14 by people with lived experience only and 17 by clinicians only (Online Resource 7). The remaining modification suggestions were identified by both groups. A full list of quotes is provided in Online Resource 8. Representative quotes are formatted as follows:

Experience type: age/child age: GMFCS level: communication ability (able to self report or complex communication needs (CCN)).

**Table 4:** Qualitative data category and subcategory description

Category	Category description	Subcategories	Further subcategories
Accessibility	Suggestions for how people with disability can access the tool to prioritise self-report	• Cognitive impairment • Complex communication needs	
Comprehensibility	Comments related to an individual’s ability to understand the tool - including but not limited to understanding the items, wording, purpose of the tool and response options	• Age • Co-occurring diagnosis • Unclear wording • Understanding what pain is	
Comprehensiveness	Suggestions to ensure the full scope of the construct is covered, as is relevant to people with cerebral palsy. The focus here is on clarifying existing items, not adding new items		
Feasibility	Suggestions related to the use of the tool in clinical practice and research by clinicians and people with lived experience	• Administration • Appropriateness of parent report • Emotional response • Purpose of the tool	*Validity of the assessment*
Presentation	How the assessment tool is displayed or presented	• Scaling • Visual presentation	
Relevance	Suggestions relating to the relevance or meaning of the tool for people with cerebral palsy	• Age group • Inappropriate wording • Meaningful to children with CP • Purpose of items • Purpose of tool • Recall period	*Autonomy*
Suggested changes	All suggested practical changes/modifications to the tools		

#### Comprehensibility

Comprehensibility, related to the ability to understand the tool itself, individual items, wording and response options. Suggestions included simplifying mBPI wording, with the FOPQ-C-SF identified as already using appropriate language. Improving comprehensibility involved using alternatives to written text, particularly for those with cognitive impairment and of younger age.

I think… with younger kids, especially non-verbal, [you should] **use a lot of visuals** because they understand through that a lot easier…. you know, or even someone who is verbal, like [my daughter], sometimes, **visuals are a lot better for her to understand** rather than asking her questions and getting her to answer.

(Parent, 9-year-old female, GMFCS II, able to self-report)

Parents and children with CP acknowledged they did not always understand what pain was because it was difficult to identify and pain was a normal part of life. Clinicians highlighted the potential difficulty for children and young people with co-occurring diagnoses of CP and Autism using a tool with abstract questions, such as the FOPQ-C-SF.

Because when I look at that question, when I feel pain I’m afraid that something terrible will happen, I just wonder how that will be interpreted…. Because it’s also got to take into account their [individuals with Autism] ability to introspect and to understand feelings as well as pain, which they may have difficulties doing.

(Psychologist)

### Accessibility

Accessibility observations primarily related to access for self-report for people with cognitive impairment and complex communication needs. Suggested improvements included using alternatives to written text, different ways to administer the tool including asking questions one at a time, having one question per page, using a Talking Mats framework [37] and providing an option for ‘I don’t know’:

So **if you are verbal**, you can say, I don’t understand what you asked me or what do you mean or you can ask questions back and forth to clarify but **if you are just expected to point to one to 10 or whatever you make the scale**, you need some of that interactive language for them to be able to say, like, **“explain this to me more”, “I don’t understand”, “I don’t know**.” Because **how do you opt out of a scale like that?**

(Speech Pathologist)

Participants also recognised making accessibility changes to the tools needed to be individualised:

Yeah, I think **simpler wording**. If we could **base it around his PODD** [it] would probably make it much easier. But then it’s kind of very customised and that’s going to take a lot of work to **specifically customise it to him**.

(Parent, 8-year-old, GMFCS V, CCN):

#### Comprehensiveness

Comprehensiveness focused on ensuring the full scope of the construct was covered as is relevant to people with CP. Suggestions included providing examples of the item with standardised examples or descriptions:

I think you would either list them all as an explainer as to what it all means or you have a separate one for each…. Daily care needs, I think, even if it’s for a mother… still needs to be specific. You need to put in brackets, washing, brushing teeth, getting dressed. I still think you need a little bit of clarification on what that means.

(Parent, GMFCS V, CCN)

Participants suggested including high frequency events specific to children and young people with CP, such as asking how pain interferes with use of assistive technology and pain related fear in the context of therapy, equipment, medical interventions or visiting health professionals.

*“When she [my daughter] sees her physiotherapist, you can just see the fear in her eyes straightaway because you know the physio is going to put her through her paces a bit and make her uncomfortable through the various stretches and exercises and all that type of thing.”*

(Parent, 9-year-old, GMFCS V, CCN)

#### Presentation

Presentation most commonly related to scaling/response options and visual presentation, closely related to accessibility and comprehensibility. All participants agreed on reducing the number of response options in the mBPI. Reducing the scale to 5 points, with visual symbols, verbal descriptions and colour scales for each of the numbers, was suggested.

Maybe one to five instead of one to 10……it’s a large scale to be choosing from… there’s not probably a lot of difference between four and five, five and six.

(39-year-old, GMFCS II)

The most commonly suggested visual symbols for the mBPI were faces showing emotions, or symbols showing empty to full. For the FOPQ-C-SF, the most commonly suggested symbol was thumbs up/thumbs down to indicate level of agreement. The importance of having a range of options for different people was highlighted, particularly having visual symbols, words and colours available together or separately on different versions. Participants also recommended larger font, bigger spacing and alternating colour rows to improve readability.

I think if you’re going to get the person with CP to fill it out though, you might want to lay it out slightly differently. Because obviously, if you’ve got trouble with fine motor skills, circling a small number is going to be quite difficult. So even if you had it in like boxes that were larger and had the wording like does not interfere and completely interferes at either end rather than one underneath and one to the side and maybe some more spacing between them would be a lot easier.

(Parent, 4-year-old, GMFCS III, able to self-report):

#### Feasibility

The feasibility of tools was identified by all participants. Suggestions related to when and how the tool is completed, appropriateness of parent report, possible emotional responses by those completing it and understanding the tool purpose. Presenting items one at a time, ensuring understanding and then selecting a response option was the preferred method. People with lived experience wanted to complete the assessments before seeing a clinician, with children preferring to complete the assessment with a parent/caregiver.

People with CP and parents both indicated a proxy report version of the FOPQ-C-SF was not appropriate because pain-related fear is too personal to be answered accurately by a proxy, even someone who knows the individual well. Pain-related fear was the only construct discussed that was identified as being too personal for a proxy report.

People with lived experience acknowledged their frustration at completing assessments without feedback from the clinician afterwards.

Because from my previous experience [they] always ask me to fill the questionnaire, **but no update afterwards**. So that’s why sometimes I just feel like, you know, they say, oh, this is very important to us, it would be great if you filled this out, blah blah blah. We do it, we take our time and we do it seriously, but sometimes **we just feel like we didn’t hear any feedback since then**. So, it would be great that everything you do for [a] purpose. **So, it would be great if I just know what’s happening**, even though maybe nothing really happened, and then you can let us know that you do it and you read it.

(Parent, 10-year-old, GMFCS II, CCN)

All groups recognised answering questions about pain could be a negative experience, highlighting the importance of clinicians knowing when a tool like this was necessary, and explaining this to the client.

#### Relevance

Relevance was identified by all participants. Alternative wording for people with disability was frequently suggested, particularly in the context of movement (i.e. using “mobility” not “walking/running”). The phrase “normal people” (item 1 FOPQ-C-SF) was seen as inappropriate for individuals with disability.

The tricky one I see straight away is, can I do all of the [things] normal people do…. because it’s easy to hurt my body? It’s not about what people can do, because… you struggle with motor skills. Some of those questions are strange, because it’ll be like, you can’t kick a ball, because your back hurts? Well, yeah, but I can’t actually kick the ball…. probably just ditch normal people. Or you can just put people. I personally don’t really care – I don’t know if I’ve been desensitised, or whatever, but I know people can get a little bit jarred with that [normal people].

(16-year-old, GMFCS II)

All groups acknowledged the FOPQ-C-SF had an assumption of autonomy and independence of the respondent, which may not be true for children with CP. Wording changes were suggested to reflect this.

[Children without disability] can move – like, if you are mobile, if someone comes at you with an injection, you bolt across the room, and you hide behind your mum, and you would be kicking and screaming. Whereas, if you can’t do that, yeah, you might be crying, but people ignore that. So, yeah, then you…start to develop…fear about all those things, because you’re not in control at all.

(Speech pathologist)

## Discussion

This study highlighted the importance of involving clinicians and people with lived experience in the modification of assessment tools for young people with CP and chronic pain. The FOPQ-C-SF and mBPI were the highest rated tools assessing pain coping and multidimensional assessment of the impact of pain on emotional functioning (respectively). Potential tool modifications were identified, relating to six categories: accessibility, comprehensibility, feasibility, relevance, presentation and comprehensiveness.

COSMIN recommends, where possible, existing assessment tools are tested and used in new populations rather than developing new tools [35]. Accordingly, we focused on validating and modifying existing tools for young people with CP. Suggestions which changed the construct of interest were not considered [35]. The eight tools included in the survey covered four main constructs: pain catastrophising, pain anxiety, pain-related fear, and pain interference with emotional functioning [9, 10, 21]. Despite high ratings of relevance, comprehensibility and feasibility across the eight tools, we chose to start by modifying the two highest-rated, which assess two constructs: fear of pain (FOPQ-C-SF) and pain interference, including pain interference with emotional functioning (mBPI). These modifications could also be applied to other future outcome measures, enabling assessment of a wider range of pain-related constructs. This study also included lived experience representation of cognitive and communication impairment to ensure accessibility of modifications.

Heterogeneity is a challenge when ensuring tools can be used in the CP population. Young people with CP can have varied motor, communication, visual, sensory and/or cognitive abilities [38]. Modifying tools to cater to all needs in the population is difficult. For example, some participants suggested adding a colour scale to improve understanding for those with cognitive impairment, whereas others disagreed because for those with visual impairment, added colours can increase confusion.

A further challenge is balancing the needs of clinicians and people with lived experience. While both groups agreed on which tools to use, differing views were held on how tools should be presented. For example, people with lived experience requested each question be displayed on a separate page, however clinicians perceived this to create additional workload by needing to collate multiple pages. Although this may be an impracticality for clinicians, modifications to improve self-report opportunity for people with lived experience should be prioritised given they are the ones completing the tools and 1 in 2 children with CP have an intellectual disability [38]. The need for adaptation in the survey was lower for the lived experience group than clinicians, despite similar numbers of changes suggested by the groups in the qualitative component. The advisory group suggested this may be because people with lived experience are used to ‘making do’ with inappropriate assessments, not because they do not need modification.

Consumer engagement is fundamental to ensure knowledge translation [39]. The advisory group was instrumental in recommending procedures to ensure representation of the heterogenous population, while reducing participant research burden. Both the quantitative and qualitative components had close to equal representation of clinicians and people with lived experience, with lived experience participants being representative of the spectrum of abilities seen in CP.

A limitation of this study was possible bias as the interviewer MGS was known to some participants. A further limitation was not interviewing children with moderate-severe cognitive impairment or complex communication needs. To gather modification suggestions, participants needed to understand the existing assessment tools and suggest changes. We instead focused on ensuring representation of these groups through proxy reporters, such as parents.

### Future directions

Identified modifications to the FOPQ-C-SF and mBPI will be further investigated to determine which are most important to implement. Further testing of comprehensibility, through pilot testing and cognitive interviews, will then take place in young people with CP and varying movement, communication and cognitive abilities. The identified modification suggestions could also be used to adapt other outcome measures for young people with CP.

## Conclusion

The FOPQ-C-SF and mBPI are the highest rated tools for assessing impact of pain on emotional functioning for children and young people with CP, provided modifications are made. Potential modifications have been identified to improve appropriateness and feasibility for children with CP and varying abilities. Future research should prioritise the involvement of people with lived experience when modifying PROMs to ensure their needs are balanced alongside those of clinicians.

### Electronic supplementary material

Below is the link to the electronic supplementary material.

Supplementary Material 1

Supplementary Material 2

Supplementary Material 3

Supplementary Material 4

Supplementary Material 5

Supplementary Material 6

Supplementary Material 7

Supplementary Material 8

Supplementary Material 9
