# Supplementary material for: Examining tools for assessing the impact of chronic pain on emotional functioning in children and young people with cerebral palsy: stakeholder preference and recommendations for modification
Source: Qual Life Res. 2024 May 25;33(8):2247–59. doi: 10.1007/s11136-024-03693-1 (PMC11286630; doi:10.1007/s11136-024-03693-1)
Supplement: Supplementary file 2 — Supplementary Material 2 [file 11136_2024_3693_MOESM2_ESM.docx]

**Supplementary material 5 – Codebook and refined coding schema**

| Category name | Description | Files | References | No. participants (/30) | Lived experience /18 | Clinicians /12 |
| --- | --- | --- | --- | --- | --- | --- |
| **Accessibility** | Suggestions for how people with disability can access the tool to prioritise self-report | 7 | 21 | 13 | 8 | 5 |
| Cognitive impairment | How to improve access to self-report for people with cognitive impairment | 5 | 6 | 6 | 3 | 3 |
| Complex communication needs | How to improve access to self-report for people with complex communication needs | 4 | 10 | 7 | 5 | 2 |
| **Comprehensibility** | Comments related to an individual's ability to understand the tool - including but not limited to understanding the items, wording, purpose of the tool and response options | 13 | 117 | 29 | 18 | 11 |
| Age | How might age impact on a person's ability to understand the assessment | 2 | 3 | 4 | 3 | 1 |
| Co-occurring diagnosis | The impact of other diagnoses (e.g. Autism) on how a person with cerebral palsy may understand the assessments | 2 | 7 | 3 | 0 | 3 |
| Unclear wording | Wording that is unclear for a person with typical cognitive ability | 7 | 17 | 19 | 9 | 10 |
| Understanding what pain is | For individuals with disability, do they understand what pain is to answer the questions? | 2 | 3 | 2 | 2 | 0 |
| **Comprehensiveness** | Suggestions to ensure the full scope of the construct is covered, as is relevant to people with cerebral palsy. The focus here is on clarifying existing items, not adding new items | 12 | 24 | 22 | 12 | 10 |
| **Feasibility** | Suggestions related to the use of the tool in clinical practise and research by clinicians and consumers | 14 | 100 | 30 | 18 | 12 |
| Administration | When and how to administer the tool | 14 | 37 | 27 | 16 | 11 |
| *Validity of the assessment* | Any factors related to administration which may impact on the validity of the assessment | 3 | 4 | 3 | 3 | 0 |
| Appropriateness of parent report | Suggestions related to whether the assessment tools can or should be completed by a proxy reporter | 5 | 11 | 13 | 12 | 1 |
| Emotional response | How the assessment itself makes participants feel or react | 8 | 16 | 16 | 11 | 5 |
| Purpose of the tool | Suggestions relating to the clinical relevance or broader purpose/use of the tool | 10 | 22 | 14 | 9 | 5 |
| **Presentation** | How the assessment tool is displayed or presented | 14 | 109 | 29 | 18 | 11 |
| Scaling | Suggestions as to how responses should be presented | 14 | 63 | 29 | 18 | 11 |
| Visual presentation | Suggestions as to how the assessment should be presented visually | 2 | 4 | 2 | 2 | 0 |
| **Relevance** | Suggestions relating to the relevance or meaning of the tool for people with cerebral palsy | 14 | 111 | 30 | 18 | 12 |
| Age group | Suggestions related to how age (e.g. younger age) might impact on the relevance of an item | 1 | 1 | 1 | 0 | 1 |
| Inappropriate wording | Wording which is inappropriate for individuals with disability, including but not limited to alternative mobility and communication strategies | 8 | 14 | 16 | 10 | 6 |
| Meaningful to children with CP | Are the tool items and response options meaningful specifically for children and young adults with cerebral palsy and their unique needs? | 9 | 12 | 18 | 10 | 8 |
| *Autonomy* | Suggestions as to how to make items more relevant for individuals with disability who may have reduced autonomy in their day to day decision making. | 9 | 12 | 18 | 10 | 8 |
| Purpose of items | Are the items themselves relevant for children and young adults with CP? | 3 | 7 | 3 | 2 | 1 |
| Purpose of tool | Is the tool as a whole relevant to children and young adults with CP? | 2 | 4 | 2 | 1 | 1 |
| Recall period | Suggestions related to the recall period of the tool (i.e. one week, two weeks, etc.) | 1 | 1 | 1 | 1 | 0 |
| **Suggested changes** | All suggested practical changes/modifications to the tools  *(see summary of suggested changes table for further detail)* | 14 | 299 | 30 | 18 | 12 |
