# Supplementary material for: Examining tools for assessing the impact of chronic pain on emotional functioning in children and young people with cerebral palsy: stakeholder preference and recommendations for modification
Source: Qual Life Res. 2024 May 25;33(8):2247–59. doi: 10.1007/s11136-024-03693-1 (PMC11286630; doi:10.1007/s11136-024-03693-1)
Supplement: Supplementary file 3 — Supplementary Material 3 [file 11136_2024_3693_MOESM3_ESM.docx]

**Supplementary material 6: Survey ratings**

TABLE 1: Pain coping tools

| Tool | Item | Agree + Strongly Agree (%)_lived_experience | Agree + Strongly Agree (%)_clinician | Disagree + Strongly Disagree (%)_lived_experience | Disagree + Strongly Disagree (%)_clinician | Agree + Strongly Agree Total (%) | Disagree + Strongly Disagree Total (%) |
| --- | --- | --- | --- | --- | --- | --- | --- |
| bapq | AAC* | 50.0 | NA | 50.0 | NA | 50.0 | 50.0 |
| cpaq | AAC* | 50.0 | NA | 50.0 | NA | 50.0 | 50.0 |
| fopq | AAC* | 50.0 | NA | 50.0 | NA | 50.0 | 50.0 |
| pcs | AAC* | 50.0 | NA | 0.0 | NA | 50.0 | 0.0 |
| pvaq | AAC* | 50.0 | NA | 50.0 | NA | 50.0 | 50.0 |
| bapq | modification | 17.2 | 78.1 | 24.1 | 6.3 | 47.7 | 15.2 |
| cpaq | modification | 10.3 | 50.0 | 20.7 | 12.5 | 30.2 | 16.6 |
| fopq | modification | 20.7 | 62.5 | 31.0 | 9.4 | 41.6 | 20.2 |
| pcs | modification | 20.7 | 53.1 | 31.0 | 3.1 | 36.9 | 17.1 |
| pvaq | modification | 13.8 | 37.5 | 34.5 | 15.6 | 25.6 | 25.1 |
| bapq | Administration* | 89.7 | 46.9 | 6.9 | 21.9 | 68.3 | 14.4 |
| cpaq | Administration* | 93.1 | 65.6 | 3.4 | 3.1 | 79.4 | 3.3 |
| fopq | Administration* | 86.2 | 75.0 | 3.4 | 0.0 | 80.6 | 1.7 |
| pcs | Administration* | 82.8 | 43.8 | 10.3 | 15.6 | 63.3 | 13.0 |
| pvaq | Administration* | 82.8 | 56.3 | 6.9 | 9.4 | 69.5 | 8.1 |
| bapq | Clinical use* | 75.9 | 46.9 | 10.3 | 25.0 | 61.4 | 17.7 |
| cpaq | Clinical use * | 86.2 | 50.0 | 10.3 | 28.1 | 68.1 | 19.2 |
| fopq | Clinical use * | 89.7 | 62.5 | 6.9 | 15.6 | 76.1 | 11.3 |
| pcs | Clinical use * | 75.9 | 25.0 | 10.3 | 34.4 | 50.4 | 22.4 |
| pvaq | Clinical use * | 82.8 | 34.4 | 3.4 | 34.4 | 58.6 | 18.9 |
| bapq | comprehensibility | 96.6 | 75.0 | 0.0 | 3.1 | 85.8 | 1.6 |
| cpaq | comprehensibility | 86.2 | 68.8 | 6.9 | 3.1 | 77.5 | 5.0 |
| fopq | comprehensibility | 93.1 | 87.5 | 3.4 | 6.3 | 90.3 | 4.8 |
| pcs | comprehensibility | 75.9 | 46.9 | 13.8 | 18.8 | 61.4 | 16.3 |
| pvaq | comprehensibility | 86.2 | 62.5 | 6.9 | 25.0 | 74.4 | 15.9 |
| bapq | Duration* | 82.8 | 75.0 | 3.4 | 3.1 | 78.9 | 3.3 |
| cpaq | Duration* | 93.1 | 96.9 | 3.4 | 0.0 | 95.0 | 1.7 |
| fopq | Duration* | 93.1 | 93.8 | 3.4 | 0.0 | 93.4 | 1.7 |
| pcs | Duration* | 79.3 | 81.3 | 3.4 | 3.1 | 80.3 | 3.3 |
| pvaq | Duration* | 82.8 | 84.4 | 3.4 | 0.0 | 83.6 | 1.7 |
| bapq | relevance | 55.2 | 40.6 | 10.3 | 18.8 | 47.9 | 14.5 |
| cpaq | relevance | 79.3 | 50.0 | 3.4 | 12.5 | 64.7 | 8.0 |
| fopq | relevance | 82.8 | 65.6 | 0.0 | 9.4 | 74.2 | 4.7 |
| pcs | relevance | 79.3 | 46.9 | 3.4 | 6.3 | 63.1 | 4.8 |
| pvaq | relevance | 75.9 | 65.6 | 3.4 | 12.5 | 70.7 | 8.0 |

*clinical feasibility was calculated as the average of AAC (for lived experience), administration, duration and clinical utility (see page 3)

**Abbreviations: bapq=**Bath Adolescent Pain Questionnaire, **cpaq=**Chronic Pain Acceptance Questionnaire, **fopq=**Fear of Pain Questionnaire for Children Short Form, **pcs=**Pain Catastrophizing Scale for Children, **pvaq=**Pain Vigilance and Awareness Questionnaire

TABLE 2: Multidimensional assessment tools

| **Tool** | **Area** | **Agree + Strongly Agree (%)_lived_experience** | **Agree + Strongly Agree (%)_clinician** | **Disagree + Strongly Disagree (%)_lived_experience** | **Disagree + Strongly Disagree (%)_clinician** | **Agree + Strongly Agree Total (%)** | **Disagree + Strongly Disagree Total (%)** |
| --- | --- | --- | --- | --- | --- | --- | --- |
| mbpi | AAC* | 50.0 | NA | 50.0 | NA | 50.0 | 50.0 |
| pbi | AAC* | 50.0 | NA | 50.0 | NA | 50.0 | 50.0 |
| ppst | AAC* | 50.0 | NA | 0.0 | NA | 50.0 | 0.0 |
| mbpi | modification | 10.3 | 34.4 | 41.4 | 25.0 | 22.4 | 33.2 |
| pbi | modification | 20.7 | 50.0 | 27.6 | 9.4 | 35.3 | 18.5 |
| ppst | modification | 27.6 | 62.5 | 31.0 | 0.0 | 45.0 | 15.5 |
| mbpi | Administration* | 93.1 | 71.9 | 3.4 | 6.3 | 82.5 | 4.8 |
| pbi | Administration* | 82.8 | 71.9 | 6.9 | 6.3 | 77.3 | 6.6 |
| ppst | Administration* | 86.2 | 84.4 | 0.0 | 3.1 | 85.3 | 1.6 |
| mbpi | Clinical use* | 89.7 | 84.4 | 6.9 | 0.0 | 87.0 | 3.4 |
| pbi | Clinical use* | 72.4 | 65.6 | 13.8 | 6.3 | 69.0 | 10.0 |
| ppst | Clinical use* | 82.8 | 53.1 | 6.9 | 18.8 | 67.9 | 12.8 |
| mbpi | comprehensibility | 82.8 | 90.6 | 10.3 | 3.1 | 86.7 | 6.7 |
| pbi | comprehensibility | 75.9 | 71.9 | 6.9 | 9.4 | 73.9 | 8.1 |
| ppst | comprehensibility | 93.1 | 96.9 | 0.0 | 0.0 | 95.0 | 0.0 |
| mbpi | Duration* | 89.7 | 90.6 | 3.4 | 0.0 | 90.1 | 1.7 |
| pbi | Duration* | 89.7 | 90.6 | 0.0 | 0.0 | 90.1 | 0.0 |
| ppst | Duration* | 86.2 | 93.8 | 0.0 | 0.0 | 90.0 | 0.0 |
| mbpi | relevance | 89.7 | 90.6 | 3.4 | 3.1 | 90.1 | 3.3 |
| pbi | relevance | 75.9 | 81.3 | 6.9 | 3.1 | 78.6 | 5.0 |
| ppst | relevance | 79.3 | 62.5 | 0.0 | 12.5 | 70.9 | 6.3 |

*clinical feasibility was calculated as the average of AAC (for lived experience), administration, duration and clinical utility (see page 3)

**Abbreviations: mbpi**=Modified Brief Pain Inventory, **pbi=**Pain Burden Inventory, **ppst=**Pediatric Pain Screening Tool

**Clinical feasibility scores – lived experience**

|  | Percentage strongly agree and agree responses | | | | |
| --- | --- | --- | --- | --- | --- |
| **Tool** | AAC | Duration | Administration | Clinical use | **Average clinical feasibility** |
| bapq | 50.0 | 82.8 | 89.7 | 75.9 | 74.6 |
| cpaq | 50.0 | 93.1 | 93.1 | 86.2 | 80.6 |
| fopq | 50.0 | 93.1 | 86.2 | 89.7 | 79.7 |
| pcs | 50.0 | 79.3 | 82.8 | 75.9 | 72 |
| pvaq | 50.0 | 82.8 | 82.8 | 82.8 | 74.6 |
| Mbpi | 50.0 | 89.7 | 93.1 | 89.7 | 80.6 |
| Pbi | 50.0 | 89.7 | 82.8 | 72.4 | 73.7 |
| ppst | 50.0 | 86.2 | 86.2 | 82.8 | 76.3 |

**Clinical feasibility scores – clinicians**

|  | Percentage strongly agree and agree responses | | | |
| --- | --- | --- | --- | --- |
| **Tool** | Duration | Administration | Clinical use | **Average clinical feasibility** |
| bapq | 75.0 | 46.9 | 46.9 | 56.2 |
| cpaq | 96.9 | 65.6 | 50.0 | 70.8 |
| fopq | 93.8 | 75.0 | 62.5 | 77.1 |
| pcs | 81.3 | 43.8 | 25.0 | 50 |
| pvaq | 84.4 | 56.3 | 34.4 | 58.3 |
| mbpi | 90.6 | 71.9 | 84.4 | 82.3 |
| pbi | 90.6 | 71.9 | 65.6 | 76.0 |
| ppst | 93.8 | 84.4 | 53.1 | 77.1 |

**Clinical feasibility scores – combined**

|  | Percentage strongly agree and agree responses | | | | |
| --- | --- | --- | --- | --- | --- |
| **Tool** | AAC | Duration | Administration | Clinical use | **Average clinical feasibility** |
| bapq | 50.0 | 78.5 | 68.3 | 61.4 | 64.6 |
| cpaq | 50.0 | 94.9 | 79.4 | 68.1 | 73.1 |
| fopq | 50.0 | 94.9 | 80.6 | 76.1 | 75.0 |
| pcs | 50.0 | 80.0 | 63.3 | 50.4 | 61.0 |
| pvaq | 50.0 | 83.3 | 69.5 | 58.6 | 65.4 |
| mbpi | 50.0 | 90.0 | 82.5 | 87.0 | 77.4 |
| pbi | 50.0 | 90.0 | 77.3 | 69.0 | 71.6 |
| ppst | 50.0 | 89.9 | 85.3 | 67.9 | 73.3 |
