# Supplementary material for: Examining tools for assessing the impact of chronic pain on emotional functioning in children and young people with cerebral palsy: stakeholder preference and recommendations for modification
Source: Qual Life Res. 2024 May 25;33(8):2247–59. doi: 10.1007/s11136-024-03693-1 (PMC11286630; doi:10.1007/s11136-024-03693-1)
Supplement: Supplementary file 4 — Supplementary Material 4 [file 11136_2024_3693_MOESM4_ESM.docx]

| **Supplementary material 8: direct quotes and their category mapping** | | | |
| --- | --- | --- | --- |
| **QUOTE** | **PARTICIPANT** | **CATEGORY** | **SUBCATEGORIES** |
| “Is that everything except what's on the other points? It just seems a bit vague. So I personally wouldn't know what fits into general activity [mBPI item 3]. I'd have to do all the others first and then come back to that one.” | Parent, 9 year old, GMFCS V, CCN | Comprehensibility | unclear wording |
| “I think… with younger kids, especially non-verbal, [you should] **use a lot of visuals** because they understand through that a lot easier….. you know, or even someone who is verbal, like [my daughter], sometimes, **visuals are a lot better for her to understand** rather than asking her questions and getting her to answer.” | Parent, 9 year old, GMFCS II, able to self-report | Comprehensibility  Accessibility | Age  Cognitive impairment |
| “I think like people said, if you put **pictures** maybe next to some of the different ones. Like if you’re sleeping in a bed, with a person sleeping in it. Activity may put like – I’m not sure, kicking a ball or something just to give them an idea of what you’re asking. For younger kids, I think that would be really helpful. **Not just younger kids, kids that have intellectual disabilities [who] are not quite [as] good with reading**. Just a visual might help them understand the question better.” | 27 year old, GMFCS I | Comprehensibility  Accessibility | Age  Cognitive impairment |
| “Yeah, pictures works really well with and so for [our son], yeah, that would be perfect to helping him understand what these questions are.” | Parent, 8 year old with CCN, GMFCS V | Comprehensibility  Accessibility | Cognitive impairment  Complex communication needs |
| “I don’t think they [children with CP] know the concept of the word, pain. The big one is heat, or, the big one is – or even bad or good. Everyone knows, or learns, eventually, that heat hurts… so, if you ask a kid, is something – not exactly hot, but if something stings – because I know, especially with cramps, it's more like an aching, or even a numb pain. So, maybe if it's more, are you feeling good or bad? Not necessarily, are you in pain? It's more, how do you feel? Or even ask them about their emotions, because, you know, [they go] hand-in-hand.” | 16 year old, GMFCS II | Comprehensibility | Understanding what pain is |
| “Because when I look at that question, when I feel pain I’m afraid that something terrible will happen, I just wonder how that will be interpreted…. Because it’s also got to take into account their [individuals with Autism] ability to introspect and to understand feelings as well as pain, which they may have difficulties doing” | Psychologist | Comprehensibility | Co-occurring diagnosis |
| “So **if you are verbal**, you can say, I don't understand what you asked me or what do you mean or you can ask questions back and forth to clarify but **if you are just expected to point to one to 10 or whatever you make the scale,** you need some of that interactive language for them to be able to say, like**, “explain this to me more”, “I don't understand”, “I don't know**.” Because **how do you opt out of a scale like that?”** | Speech Pathologist | Accessibility | Complex communication needs |
| “Yeah, I think **simpler wording**. If we could **base it around his PODD** [it] would probably make it much easier. But then it’s kind of very customised and that’s going to take a lot of work to **specifically customise it to him**.” | Parent, 8 year old, GMFCS V, CCN | Accessibility | Complex communication needs |
| “I think you would either list them all as an explainer as to what it all means or you have a separate one for each….. Daily care needs, I think, even if it's for a mother… still needs to be specific. You need to put in brackets, washing, brushing teeth, getting dressed. I still think you need a little bit of clarification on what that means” | Parent, GMFCS V, CCN | Comprehensiveness  Comprehensibility | Unclear wording |
| “When she [my daughter] sees her physiotherapist, you can just see the fear in her eyes straightaway because you know the physio is going to put her through her paces a bit and make her uncomfortable through the various stretches and exercises and all that type of thing.” | Parent, 9 year old, GMFCS V, CCN | Comprehensiveness  Relevance | Meaningful to children with CP |
| “Maybe something along the lines, I avoid using particular equipment. It could be, I avoid putting my AFO (ankle foot orthotic) on, or I avoid using a particular walking aid for example, because it might make my pain worse” | Psychologist | Comprehensiveness  Relevance | Meaningful to children with CP |
| “The big one is probably the outcome, if pain gets better or worse, with physical activity. Because I know…. the big treatment I have that tends to help sometimes is I've [got a] heated my pool at my house, so, when I go for a swim, my back feels a lot better….The big one is, you don’t want kids to be in severe pain after physio, but after physio, if you're feeling better than you were, that means you’ve been doing the right thing.” | 16 year old, GMFCS II | Comprehensiveness  Relevance | Meaningful to children with CP |
| “Maybe one to five instead of one to 10……it's a large scale to be choosing from… there's not probably a lot of difference between four and five, five and six” | 39 year old, GMFCS II | Presentation  Comprehensibility | Scaling  Unclear wording |
| “I can't figure out what the [difference] between the one and the two might be. Because if it's affecting my sleep, then it's affecting my sleep or affecting my child's sleep. I can't see that it's affecting it point three of a - a tenth of what it should be. So literally then it would be like nine steps to a good sleep, you know” | Parent, GMFCS V, CCN | Presentation  Comprehensibility | Scaling  Unclear wording |
| “I think we’re all – I think we’re a bit complacent with the zero to 10. I think for me, my initial reaction is, yeah, it’s fine. But I think sometimes when we do these…. what does six actually mean?” | 28 year old, GMFCS IV, able to self-report | Presentation  Comprehensibility | Scaling  Unclear wording |
| “You could do colours, so zero being red, five being orange, a 10 being green and that way he [my son] could probably be able to work it to tell us where he is on that” | Parent, 8 year old, GMFCS V, CCN | Presentation  Accessibility | Scaling  Cognitive impairment |
| “I've usually done, yeah, the faces. Like number one is a smiling face, then number two is a little bit more worried, and then up to number five, where… the symbol is called angry or frustrated, or something, but it's like there's steam coming to their ears, and they're really like grasping their [head]. It's sort of that level of difference. Then I also colour code it. So, the background of one would be green, and then all the way down to red.” | Speech pathologist | Presentation | Scaling |
| “Yeah, just agree, strongly agree… so, like, agree could one thumbs up, strongly agree could be two thumbs up. Strongly disagree could be two down, one down. Unsure could be [thumbs sideways]” | 28 year old, GMFCS IV, able to self-report | Presentation | Scaling |
| “I also wonder if it's worth having different versions. So for example, one of them is all smiley face…. One is more of a coloured scale. Because people respond differently to different methods. So they've got the choice of what works best for them but it's still recounting the same information and we can just choose which version matches that client best.” | Physiotherapist | Presentation  Feasibility | Visual presentation  Administration |
| “I think if you're going to get the person with CP to fill it out though, you might want to lay it out slightly differently. Because obviously, if you've got trouble with fine motor skills, circling a small number is going to be quite difficult. So even if you had it in like boxes that were larger and had the wording like does not interfere and completely interferes at either end rather than one underneath and one to the side and maybe some more spacing between them would be a lot easier” | Parent, 4 year old, GMFCS III, able to self-report | Presentation  Accessibility | Visual presentation |
| “Can you do them [questions] **one at a time** next time please?” | 15 year old, GMFCS II | Feasibility  Accessibility | Administration  Cognitive impairment |
| “I guess parents observe their children and **can have some idea of the level of pain** they're in but children may also be **struggling to communicate** and actually **realise that they're in pain** as much as they are because they don't know any different. So, yeah, I guess that's something I found myself, both throughout childhood and as an adult, **you don't know any different**.” | 39 year old, GMFCS II | Feasibility | Appropriateness of parent report |
| “It’s **all a guessing game** for how [he] would be feeling with the questions being asked of him.” | Parent, 8 year old, GMFCS V, CCN | Feasibility | Appropriateness of parent report |
| “I'd be **speculating on pretty much the whole lot** and, like the point, it is - it's all relative and, you know, my partner and I, we've often wondered… does [our daughter] know.. this or that **when she's never seen the other side**.” | Parent, 9 year old, GMFCS V, CCN | Feasibility | Appropriateness of parent report |
| “I don't know that he's [my son] afraid of pain. I don't know what pain he feels. We don't do anything because of his high medical condition. So none of this would apply to me….there should be a not applicable category then where someone can actually then write the reasons why you can't say because [otherwise] it’d all be unsure and then it wouldn't be clear why it would be unsure for me. I would have no idea because I can't communicate with my son*”* | Parent, GMFCS V, CCN | Feasibility | Appropriateness of parent report |
| “Because from my previous experience always ask me to fill the questionnaire, **but no update afterwards**. So that’s why sometimes I just feel like, you know, they say, oh, this is very important to us, it would be great if you filled this out, blah blah blah. We do it, we take our time and we do it seriously, but sometimes **we just feel like we didn’t hear any feedback since then**. So, it would be great that everything you do for purpose. **So, it would be great if I just know what’s happening**, even though maybe nothing really happened, and then you can let us know that you do it and you read it” | Parent, 10 year old, GMFCS II, CCN | Feasibility  Relevance | Purpose of tool  Purpose of tool |
| “You need to make sure you've got your intent right, as to what's the actual purpose of me doing this tool and being really clear and meaningful about what it is and what you're going to get out of it. Rather than be like oh great, another tool I can use just to do a quick screen and capture with my clients. That's all well and good but it's not necessarily being client centred” | Physiotherapist | Feasibility | Emotional response |
| “Mobility covers everyone, like even if you are in a wheelchair, you might be head switching but that's movement for you” | Physiotherapist) | Relevance | Inappropriate wording  Meaningful to children with CP |
| “The tricky one I see straight away is, can I do all of the normal people do…. because it's easy to hurt my body? It’s not about what people can do, because… you struggle with motor skills. Some of those questions are strange, because it’ll be like, you can't kick a ball, because your back hurts? Well, yeah, but I can't actually kick the ball…. probably just ditch normal people. Or you can just put people. I personally don’t really care – I don’t know if I've been desensitised, or whatever, but I know people can get a little bit jarred with that [normal people].” | 16 year old, GMFCS II | Relevance | Inappropriate wording |
| “But there are so many other factors that would mean that they have to go to school because parents are working or - it's not that the child has the complete control over why they have to go to school even though they would feel that their pain is at a level that will stop them from going to school that's not always the reason why they have to go to school.” | Parent, GMFCS V, CCN | Relevance | Meaningful to children with CP - autonomy |
| “I used to go to school all the time because my parents were quite, you have to go to school. But when I was at school, it was quite hard because I used to fall all the time. I had lots of falls because I am quite mobile so I didn't have any aids or anything.” | 30 year old, GMFCS III | Relevance | Meaningful to children with CP - autonomy |
| “It's interesting too, this assumes quite a lot of agency – that the child has quite a lot of agency. So, like, I stop any activity if I start to hurt, or my pain becomes worse. I avoid making plans… I've watched [therapy] where kids could be screaming, but they are pushed through that…. It kind of implies that the child is in control of that, that they're able to communicate that really clearly.” | Speech pathologist | Relevance | Meaningful to children with CP - autonomy |
| “[Children without disability] can move – like, if you are mobile, if someone comes at you with an injection, you bolt across the room, and you hide behind your mum, and you would be kicking and screaming. Whereas, if you can't do that, yeah, you might be crying, but people ignore that. So, yeah, then you…start to develop….fear about all those things, because you're not in control at all” | Speech pathologist | Relevance | Meaningful to children with CP - autonomy |
| “I think my problem is focusing sometimes at school…. Because the pain’s distracting me.” | 12 year old, GMFCS II, able to self-report | Relevance | Meaningful to children with CP |
| “It really hurts when I'm trying to play with someone, trying contact sports. Trying to talk to them, every time, it’s really sore…. [the pain feels] very unfair on me. It’s really unfair on me because not a lot of people ask me to join in their game.” | 15 year old, GMFCS II, able to self-report | Relevance | Meaningful to children with CP |
