# Supplementary material for: Examining tools for assessing the impact of chronic pain on emotional functioning in children and young people with cerebral palsy: stakeholder preference and recommendations for modification
Source: Qual Life Res. 2024 May 25;33(8):2247–59. doi: 10.1007/s11136-024-03693-1 (PMC11286630; doi:10.1007/s11136-024-03693-1)
Supplement: Supplementary file 5 — Supplementary Material 5 [file 11136_2024_3693_MOESM5_ESM.pdf]

Chronic Pain Assessment In Cerebral Palsy

You are eligible to participate in this study.

Welcome to the online survey.

Survey instructions:

In this round, you will be presented with 8 chronic pain assessment tools which could be used in cerebral palsy. We have focused on tools which assess the impact of pain on emotional wellbeing. The tools have been divided into 'screening' tools and more in-depth 'measurement' tools.

For each assessment tool/questionnaire, a picture of the tool has been provided along with some information on how the tool is completed, time it takes, etc.

Please read the tool (you do not need to complete the tool itself) and then answer the 5-6 multiple choice questions about the tool's relevance and ease of use.

We estimate that this survey will take approximately 20 minutes to complete.

The 'save and return later' button can be used if needed.

A note for individuals with lived experience of cerebral palsy:

Pain in cerebral palsy is generally not well understood or assessed. We would like your input as someone with lived experience of cerebral palsy to help us work out which of the following pain assessments could be relevant and meaningful. We hope that improving chronic pain assessment will in turn improve access to chronic pain treatment for people with cerebral palsy.

We know that many of the assessment tools we are presenting are not perfect and that they need adapting. We really value your input in suggesting which of these tools are most appropriate and how they could be improved.

Accessibility: this survey has a voice to text option if this is helpful

The first 5 tools could be used as a more in-depth measure of the impact of chronic pain on emotional wellbeing.

Please look at each of them individually and answer the questions below.

The Bath Adolescent Pain Questionnaire

Time to complete Approximately 20 minutes
Availability Freely available on the internet
How to complete Written or verbal questionnaire with parent-report and adolescent-report version

It has 7 subscales - we have included 1-5 for you to consider, but the full version is here

Who is it for? Adolescents aged 11-18 years
What aspects of pain does it assess? Impact of pain on: Emotional wellbeing Physical ability Participation Quality of life

Sample of the Bath Adolescent Pain Questionnaire:

you do not need answer this questionnaire, just read it and scroll down to 'questions start here' to answer the survey questions

---

Questions start here:

The Bath Adolescent Pain Questionnaire items are relevant and meaningful to people with cerebral palsy

◯ Strongly agree
◯ Agree
◯ Neutral
◯ Disagree
◯ Strongly disagree

The item and response option wording is clear and easy to understand

◯ Strongly agree
◯ Agree
◯ Neutral
◯ Disagree
◯ Strongly disagree

The time the Bath Adolescent Pain Questionnaire takes to complete is feasible

◯ Strongly agree
◯ Agree
◯ Neutral
◯ Disagree
◯ Strongly disagree

The way the Bath Adolescent Pain Questionnaire is administered (written questionnaire, interview etc.) is appropriate and feasible

◯ Strongly agree
◯ Agree
◯ Neutral
◯ Disagree
◯ Strongly disagree

The Bath Adolescent Pain Questionnaire could be used with my communication device

◯ Strongly agree
◯ Agree
◯ Neutral
◯ Disagree
◯ Strongly disagree

The Bath Adolescent Pain Questionnaire could be used with my child's communication device

◯ Strongly agree
◯ Agree
◯ Neutral
◯ Disagree
◯ Strongly disagree

The Bath Adolescent Pain Questionnaire requires adaptation to be appropriate for people with cerebral palsy

◯ Strongly agree
◯ Agree
◯ Neutral
◯ Disagree
◯ Strongly disagree

What adaptations would you suggest?

____
(optional)

I would consider using Bath Adolescent Pain Questionnaire in clinical practice

◯ Strongly agree
◯ Agree
◯ Neutral
◯ Disagree
◯ Strongly disagree

I would be happy to complete the Bath Adolescent Pain Questionnaire as part of an assessment

◯ Strongly agree
◯ Agree
◯ Neutral
◯ Disagree
◯ Strongly disagree

Any additional comments?

____

1/8 complete

.

Tool 2: Chronic Pain Acceptance Questionnaire - Adolescent

Time to complete Approximately 3-5 minutes
Availability Freely available on the internet
How to complete Written or verbal questionnaire with parent-report and adolescent-report version
Who is it for? Adolescents
What aspects of pain does it assess? Impact of pain on: Emotional wellbeing Physical ability Participation Quality of life

Sample of the Chronic Pain Acceptance Questionnaire - 8 item version:

Questions start here:

The Chronic Pain Acceptance Questionnaire items/questions are relevant and meaningful to people with cerebral palsy

◯ Strongly agree
◯ Agree
◯ Neutral
◯ Disagree
◯ Strongly disagree

The item and response option wording is clear and easy to understand

◯ Strongly agree
◯ Agree
◯ Neutral
◯ Disagree
◯ Strongly disagree

The time the Chronic Pain Acceptance Questionnaire takes to complete is feasible

◯ Strongly agree
◯ Agree
◯ Neutral
◯ Disagree
◯ Strongly disagree

The way the Chronic Pain Acceptance Questionnaire is administered (written questionnaire, interview etc.) is appropriate and feasible

◯ Strongly agree
◯ Agree
◯ Neutral
◯ Disagree
◯ Strongly disagree

The Chronic Pain Acceptance Questionnaire could be used with my communication device

◯ Strongly agree
◯ Agree
◯ Neutral
◯ Disagree
◯ Strongly disagree

The Chronic Pain Acceptance Questionnaire could be used with my child's communication device

◯ Strongly agree
◯ Agree
◯ Neutral
◯ Disagree
◯ Strongly disagree

The Chronic Pain Acceptance Questionnaire requires adaptation to be appropriate for people with cerebral palsy

◯ Strongly agree
◯ Agree
◯ Neutral
◯ Disagree
◯ Strongly disagree

What adaptations would you suggest?

____
(optional)

I would consider using the Chronic Pain Acceptance Questionnaire in clinical practice

◯ Strongly agree
◯ Agree
◯ Neutral
◯ Disagree
◯ Strongly disagree

I would be happy to complete the Chronic Pain Acceptance Questionnaire as part of an assessment

◯ Strongly agree
◯ Agree
◯ Neutral
◯ Disagree
◯ Strongly disagree

Any additional comments?

____

2/8

.

Tool 3: Fear of Pain Questionnaire for Children

Time to complete Approximately 5 minutes
Availability Freely available on the internet
How to complete Written or verbal questionnaire with parent and child report versions
Who is it for? Children, adolescents and young adults (8-25 years)
What aspects of pain does it assess? Impact of pain on: Emotional wellbeing Physical ability Participation

Sample of the Fear of Pain Questionnaire for Children - short form:

Questions start here:

The Fear of Pain Questionnaire items/questions are relevant and meaningful to people with cerebral palsy

◯ Strongly agree
◯ Agree
◯ Neutral
◯ Disagree
◯ Strongly disagree

The item and response option wording is clear and easy to understand

◯ Strongly agree
◯ Agree
◯ Neutral
◯ Disagree
◯ Strongly disagree

The time the Fear of Pain Questionnaire takes to complete is feasible

◯ Strongly agree
◯ Agree
◯ Neutral
◯ Disagree
◯ Strongly disagree

The way the Fear of Pain Questionnaire is administered (written questionnaire, interview etc.) is appropriate and feasible

◯ Strongly agree
◯ Agree
◯ Neutral
◯ Disagree
◯ Strongly disagree

The Fear of Pain Questionnaire could be used with my communication device

◯ Strongly agree
◯ Agree
◯ Neutral
◯ Disagree
◯ Strongly disagree

The Fear of Pain Questionnaire could be used with my child's communication device

◯ Strongly agree
◯ Agree
◯ Neutral
◯ Disagree
◯ Strongly disagree

The Fear of Pain Questionnaire requires adaptation to be appropriate for people with cerebral palsy

◯ Strongly agree
◯ Agree
◯ Neutral
◯ Disagree
◯ Strongly disagree

What adaptations would you suggest?

____
(optional)

I would consider using the Fear of Pain Questionnaire in clinical practice

◯ Strongly agree
◯ Agree
◯ Neutral
◯ Disagree
◯ Strongly disagree

I would be happy to complete the Fear of Pain Questionnaire as part of an assessment

◯ Strongly agree
◯ Agree
◯ Neutral
◯ Disagree
◯ Strongly disagree

Any additional comments?

____

3/8 complete

.

Tool 4: The Pain Catastrophizing Scale for Children

Time to complete 5-10 minutes
Availability Freely available on the internet
How to complete Written or verbal questionnaire with parent-report and child-report versions
Who is it for? Adolescents
What aspects of pain does it assess? Impact of pain on: Emotional wellbeing

Sample of the Pain Catastrophizing Scale for Children:

Questions start here:

The Pain Catastrophizing Scale items/questions are relevant and meaningful to people with cerebral palsy

◯ Strongly agree
◯ Agree
◯ Neutral
◯ Disagree
◯ Strongly disagree

The item and response option wording is clear and easy to understand

◯ Strongly agree
◯ Agree
◯ Neutral
◯ Disagree
◯ Strongly disagree

The time the Pain Catastrophizing Scale takes to complete is feasible

◯ Strongly agree
◯ Agree
◯ Neutral
◯ Disagree
◯ Strongly disagree

The way the Pain Catastrophizing Scale is administered (written questionnaire, interview etc.) is appropriate and feasible

◯ Strongly agree
◯ Agree
◯ Neutral
◯ Disagree
◯ Strongly disagree

The Pain Catastrophizing Scale could be used with my communication device

◯ Strongly agree
◯ Agree
◯ Neutral
◯ Disagree
◯ Strongly disagree

The Pain Catastrophizing Scale could be used with my child's communication device

◯ Strongly agree
◯ Agree
◯ Neutral
◯ Disagree
◯ Strongly disagree

The Pain Catastrophizing Scale requires adaptation to be appropriate for people with cerebral palsy

◯ Strongly agree
◯ Agree
◯ Neutral
◯ Disagree
◯ Strongly disagree

What adaptations would you suggest?

____
(optional)

I would consider using the Pain Catastrophizing Scale in clinical practice

◯ Strongly agree
◯ Agree
◯ Neutral
◯ Disagree
◯ Strongly disagree

I would be happy to complete the Pain Catastrophizing Scale as part of an assessment

◯ Strongly agree
◯ Agree
◯ Neutral
◯ Disagree
◯ Strongly disagree

Any additional comments?

____

4/8 complete

.

Tool 5: Pain Vigilance & Awareness Questionnaire

Time to complete 10 minutes
Availability Freely available on the internet
How to complete Written or verbal questionnaire, child report only
Who is it for? Children & adolescents (8-18 years)
What aspects of pain does it assess? Impact of pain on: Emotional wellbeing

Sample of the Pain Vigilance and Awareness Questionnaire:

Questions start here:

The Pain Vigilance & Awareness Questionnaire items/questions are relevant and meaningful to people with cerebral palsy

◯ Strongly agree
◯ Agree
◯ Neutral
◯ Disagree
◯ Strongly disagree

The item and response option wording is clear and easy to understand

◯ Strongly agree
◯ Agree
◯ Neutral
◯ Disagree
◯ Strongly disagree

The time the Pain Vigilance & Awareness Questionnaire takes to complete is feasible

◯ Strongly agree
◯ Agree
◯ Neutral
◯ Disagree
◯ Strongly disagree

The way the Pain Vigilance & Awareness Questionnaire is administered (written questionnaire, interview etc.) is appropriate and feasible

◯ Strongly agree
◯ Agree
◯ Neutral
◯ Disagree
◯ Strongly disagree

The Pain Vigilance & Awareness Questionnaire could be used with my communication device

◯ Strongly agree
◯ Agree
◯ Neutral
◯ Disagree
◯ Strongly disagree

The Pain Vigilance & Awareness Questionnaire could be used with my child's communication device

◯ Strongly agree
◯ Agree
◯ Neutral
◯ Disagree
◯ Strongly disagree

The Pain Vigilance & Awareness Questionnaire requires adaptation to be appropriate for people with cerebral palsy

◯ Strongly agree
◯ Agree
◯ Neutral
◯ Disagree
◯ Strongly disagree

What adaptations would you suggest?

____
(optional)

I would consider using the Pain Vigilance & Awareness Questionnaire in clinical practice

◯ Strongly agree
◯ Agree
◯ Neutral
◯ Disagree
◯ Strongly disagree

I would be happy to complete the Pain Vigilance & Awareness Questionnaire as part of an assessment

◯ Strongly agree
◯ Agree
◯ Neutral
◯ Disagree
◯ Strongly disagree

Any additional comments?

____

5/8 complete

.

The last 3 tools could be used to screen the impact of chronic pain on emotional wellbeing as well as other areas. They are shorter than the first 5 tools.

Please look at each of them individually and answer the questions below.

The Modified Brief Pain Inventory   Time to complete 5 minutes
Availability Through a journal article
How to complete Written or verbal questionnaire with proxy report and self-report option
Who is it for? Children, adolescents and young adults
What aspects of pain does it assess? Impact of pain on:

Emotional wellbeing Physical ability Participation Quality of life Sleep

Sample of the Modified Brief Pain Inventory:

Questions start here:

The Modified Brief Pain Inventory items/questions are relevant and meaningful to people with cerebral palsy

◯ Strongly agree
◯ Agree
◯ Neutral
◯ Disagree
◯ Strongly disagree

The item and response option wording is clear and easy to understand

◯ Strongly agree
◯ Agree
◯ Neutral
◯ Disagree
◯ Strongly disagree

The time the Modified Brief Pain Inventory takes to complete is feasible

◯ Strongly agree
◯ Agree
◯ Neutral
◯ Disagree
◯ Strongly disagree

The way the Modified Brief Pain Inventory is administered (written questionnaire, interview etc.) is appropriate and feasible

◯ Strongly agree
◯ Agree
◯ Neutral
◯ Disagree
◯ Strongly disagree

The Modified Brief Pain Inventory could be used with my communication device

◯ Strongly agree
◯ Agree
◯ Neutral
◯ Disagree
◯ Strongly disagree

The Modified Brief Pain Inventory could be used with my child's communication device

◯ Strongly agree
◯ Agree
◯ Neutral
◯ Disagree
◯ Strongly disagree

The Modified Brief Pain Inventory requires adaptation to be appropriate for people with cerebral palsy

◯ Strongly agree
◯ Agree
◯ Neutral
◯ Disagree
◯ Strongly disagree

What adaptations would you suggest?

____
(optional)

I would consider using the Modified Brief Pain Inventory in clinical practice

◯ Strongly agree
◯ Agree
◯ Neutral
◯ Disagree
◯ Strongly disagree

I would be happy to complete the Modified Brief Pain Inventory as part of an assessment

◯ Strongly agree
◯ Agree
◯ Neutral
◯ Disagree
◯ Strongly disagree

Any additional comments?

____

6/8 complete

.

Tool 6: Pain Burden Inventory

Time to complete 5 minutes
Availability Through a journal article
How to complete Written or verbal questionnaire, self and parent report
Who is it for? Children, adolescents and young adults (7-21 years)
What aspects of pain does it assess? Pain frequency Impact of pain on:

Emotional wellbeing Physical ability Participation Sleep

Sample of the Pain Burden Inventory:

Questions start here:

The Pain Burden Inventory items/questions are relevant and meaningful to people with cerebral palsy

◯ Strongly agree
◯ Agree
◯ Neutral
◯ Disagree
◯ Strongly disagree

The item and response option wording is clear and easy to understand

◯ Strongly agree
◯ Agree
◯ Neutral
◯ Disagree
◯ Strongly disagree

The time the Pain Burden Inventory takes to complete is feasible

◯ Strongly agree
◯ Agree
◯ Neutral
◯ Disagree
◯ Strongly disagree

The way the Pain Burden Inventory is administered (written questionnaire, interview etc.) is appropriate and feasible

◯ Strongly agree
◯ Agree
◯ Neutral
◯ Disagree
◯ Strongly disagree

The Pain Burden Inventory could be used with my communication device

◯ Strongly agree
◯ Agree
◯ Neutral
◯ Disagree
◯ Strongly disagree

The Pain Burden Inventory could be used with my child's communication device

◯ Strongly agree
◯ Agree
◯ Neutral
◯ Disagree
◯ Strongly disagree

The Pain Burden Inventory requires adaptation to be appropriate for people with cerebral palsy

◯ Strongly agree
◯ Agree
◯ Neutral
◯ Disagree
◯ Strongly disagree

What adaptations would you suggest?

____
(optional)

I would consider using the Pain Burden Inventory in clinical practice

◯ Strongly agree
◯ Agree
◯ Neutral
◯ Disagree
◯ Strongly disagree

I would be happy to complete the Pain Burden Inventory as part of an assessment

◯ Strongly agree
◯ Agree
◯ Neutral
◯ Disagree
◯ Strongly disagree

Any additional comments?

____

7/8 complete

.

Tool 7: Pediatric Pain Screening Tool

Time to complete 5 minutes
Availability Freely available
How to complete Written or verbal questionnaire, self report only
Who is it for? Children & adolescents (8-18 years)
What aspects of pain does it assess? Pain location Pain frequency Impact of pain on:

Emotional wellbeing Physical ability Participation Quality of Life Sleep

Sample of the Pediatric Pain Screening Tool:

Questions start here:

The Pediatric Pain Screening Tool items/questions are relevant and meaningful to people with cerebral palsy

- ◯ Strongly agree
- ◯ Agree
- ◯ Neutral
- ◯ Disagree
- ◯ Strongly disagree

The item and response option wording is clear and easy to understand

- ◯ Strongly agree
- ◯ Agree
- ◯ Neutral
- ◯ Disagree
- ◯ Strongly disagree

The time the Pediatric Pain Screening Tool takes to complete is feasible

- ◯ Strongly agree
- ◯ Agree
- ◯ Neutral
- ◯ Disagree
- ◯ Strongly disagree

The way the Pediatric Pain Screening Tool is administered (written questionnaire, interview etc.) is appropriate and feasible

- ◯ Strongly agree
- ◯ Agree
- ◯ Neutral
- ◯ Disagree
- ◯ Strongly disagree

The Pediatric Pain Screening Tool could be used with my communication device

- ◯ Strongly agree
- ◯ Agree
- ◯ Neutral
- ◯ Disagree
- ◯ Strongly disagree

The Pediatric Pain Screening Tool could be used with my child's communication device

◯ Strongly agree
◯ Agree
◯ Neutral
◯ Disagree
◯ Strongly disagree

The Pediatric Pain Screening Tool requires adaptation to be appropriate for people with cerebral palsy

◯ Strongly agree
◯ Agree
◯ Neutral
◯ Disagree
◯ Strongly disagree

What adaptations would you suggest?

____
(optional)

I would consider using the Pediatric Pain Screening Tool in clinical practice

◯ Strongly agree
◯ Agree
◯ Neutral
◯ Disagree
◯ Strongly disagree

I would be happy to complete the Pediatric Pain Screening Tool as part of an assessment

◯ Strongly agree
◯ Agree
◯ Neutral
◯ Disagree
◯ Strongly disagree

Any additional comments?

____

8/8 complete

.

Finishing off

Would you be willing and able to participate in a 30-60 minute online discussion (focus group or interview) to suggest modifications to these assessment tools?<br><br>You will receive a $50 gift voucher for your time	◯ Yes<br>◯ No
Do you have a child who would be willing and able to participate in a 30-60 minute online discussion (focus group or interview to suggest modifications to these assessment tools?)<br><br>They will receive a $50 gift voucher for their time	◯ Yes<br>◯ No

Thankyou for completing the survey. Please press "submit" to finish
