# Supplementary material for: Examining tools for assessing the impact of chronic pain on emotional functioning in children and young people with cerebral palsy: stakeholder preference and recommendations for modification
Source: Qual Life Res. 2024 May 25;33(8):2247–59. doi: 10.1007/s11136-024-03693-1 (PMC11286630; doi:10.1007/s11136-024-03693-1)
Supplement: Supplementary file 6 — Supplementary Material 6 [file 11136_2024_3693_MOESM6_ESM.docx]

**Supplementary material 7 – modification suggestions mapped to big picture categories**

| Suggested changes and modifications to the tools | Accessibility | Comprehensibility | Comprehensiveness | Feasibility | Presentation | Relevance | Clinicians | Lived experience | Total participants | References |
| --- | --- | --- | --- | --- | --- | --- | --- | --- | --- | --- |
| 1. **Add visual symbols for each item** | X | X |  |  | X | X | X | X | 10 | 22 |
| 1. Could use Pragmatic Organised Dynamic Display (PODD) symbols |  |  |  |  | X |  |  | X | 1 | 1 |
| 1. Modified Brief Pain Inventory (mBPI) |  |  |  |  |  |  | X | X | 4 | 5 |
| *Include meal times as one of the visuals for 'taking care of daily needs'* |  |  |  |  |  |  | X |  | 1 | 1 |
| *General activity- moving around pictures* |  |  |  |  | X |  | X |  | 1 | 1 |
| *Recreational activities- pictures of outdoor activities and sports* |  | X |  |  |  | X |  | X | 2 | 3 |
| 1. **Administration instructions** | X | X |  | X | X |  | X | X | 21 | 34 |
| 1. Clinicians must feedback the results to the client or family |  |  |  | X | X |  | X | X | 3 | 3 |
| 1. Clinicians to explain what the assessment is used for |  |  |  | X |  |  | X | X | 4 | 5 |
| 1. Complete prior to an appointment |  |  |  | X |  |  | X | X | 10 | 8 |
| 1. Complete with a support person or caregiver |  |  |  | X |  |  |  | X | 7 | 6 |
| 1. Every person with CP should be given the Fear of Pain Questionnaire for children (FOPQ-C) as they might not know that their pain is related to fear |  |  |  | X |  |  |  | X | 1 | 1 |
| 1. Have an option for audio | X | X |  | X | X |  | X | X | 3 | 3 |
| 1. For clinicians, links to other tools you could use to assess aspects of pain interference and coping further |  |  |  | X |  |  | X |  | 2 | 1 |
| 1. Present questions one at a time | X | X |  | X | X |  |  | X | 4 | 4 |
| 1. Set of quick instructions for users | x | x |  | x |  |  | x |  | 3 | 3 |
| 1. **Changes to wording** | X | X | X | X | X | X | X | X | 29 | 130 |
| 1. Add wording options for children younger than school age |  |  |  | X |  |  | X |  | 1 | 1 |
| 1. Change words which assume autonomy to 'I wish I could' or 'I want to' | X | X | X |  | X | X | X | X | 10 | 11 |
| 1. Fear of Pain Questionnaire for Children (FOPQ-C) |  | X | X | X | X | X | X | X | 20 | 34 |
| *Add other anxiety symptoms to 'pain causes my heart to beat fast'* |  | X |  |  |  | X |  | X | 5 | 5 |
| *Add standardised examples or descriptions* |  |  |  | X |  |  | X | X | 2 | 2 |
| *Change 'I cancel plans' to ‘I don’t want to do things’ or ‘I don’t want to go to things’* |  | X |  |  |  | X | X |  | 1 | 2 |
| *Change 'I hurt' to 'When I hurt'* |  |  |  |  |  |  | X |  | 1 | 1 |
| *Change 'I stop doing things when I'm in pain' to 'pain stops me doing things I want to do'* |  | X |  |  |  |  | X |  | 1 | 1 |
| *Change order of questions* |  |  |  |  | X |  | X |  | 5 | 3 |
| *Change wording from ‘feelings of pain are scary for me’ to 'I'm scared to do XXXX because of my pain'* |  |  | X |  |  |  | X |  | 2 | 1 |
| *Change wording from negative to positive* |  |  |  | X | X |  | X |  | 1 | 2 |
| *Clarify what 'something terrible' is* |  | X |  |  | X | X | X | X | 5 | 5 |
| *Combine ‘put things off’ and*  *‘avoid making plans’* |  |  |  |  | X | X |  | X | 1 | 1 |
| *Remove the word ‘normal’ from item 1 and simplify the wording* |  | X |  | X |  | X | X | X | 9 | 11 |
| 1. Make the wording of each item simpler | X |  |  |  |  |  |  | X | 2 | 1 |
| 1. Modified Brief Pain Inventory (mBPI) | X | X | X | X | X | X | X | X | 28 | 84 |
| *Add standardised examples or descriptions to each item* |  | X | X | X | X | X | X | X | 18 | 27 |
| *Change 'communication with others' to 'tell people what I'd like to tell them''* |  | X |  | X | X |  | X | X | 6 | 4 |
| *Change 'general activity' to 'getting around'* |  | X |  |  |  | X | X | X | 10 | 9 |
| *Change 'interferes' to 'gets in the way of' or 'stops me'* | X | X |  |  | X |  | X | X | 9 | 8 |
| *Change 'learning new information or skills' to 'learning new things' or 'concentration'* |  | X |  |  |  |  | X |  | 4 | 3 |
| *Change ‘mood’ to 'feelings'* | X | X |  |  |  |  | X | X | 8 | 6 |
| *Change 'recreational activity' to 'play' or 'things I do for fun'* |  | X |  |  |  | X | X | X | 14 | 10 |
| *Change 'relations with others' to 'friendships' or 'playing with my friends' or 'getting along with others' or 'spending time with friends and family'* |  | X |  |  |  | X | X | X | 12 | 9 |
| *Change 'taking care of daily needs' to 'looking after myself'* |  | X |  |  |  | X | X | X | 2 | 2 |
| *Consider leaving out 'enjoyment of life' for a child or changing to ‘having fun’* |  | X |  |  | X | X | X | X | 6 | 6 |
| 1. **Have different versions of the tool for people with different needs** | X | X |  | X | X |  | X | X | 4 | 4 |
| Electronic and printable versions of the tool | X |  |  | X | X |  | X |  | 1 | 3 |
| 1. **Items that could be added** |  |  | X | X | X | X | X | X | 16 | 19 |
| 1. Fear of Pain Questionnaire for Children (FOPQ-C) |  |  | X | X | X | X | X | X | 10 | 12 |
| *Add a comments section* |  |  |  |  | X |  | X | X | 2 | 2 |
| *Add 'fear of therapy, equipment, medical intervention, doctor'* |  |  | X | X |  | X | X | X | 9 | 9 |
| *Specify not mobilising or moving because of pain* |  |  | X |  |  |  | X |  | 1 | 1 |
| 1. Modified Brief Pain Inventory (mBPI) |  |  | X |  |  | X | X | X | 6 | 7 |
| *Add a blank option for favourite activity* |  |  | X |  |  | X | X | X | 2 | 2 |
| *Add a prompt or comment about how pain interferes with ability to use equipment and assistive technology* |  |  | X |  |  |  |  | X | 1 | 1 |
| *Add 'is there a time when you are pain free?' comment* |  |  | X |  |  |  | X | X | 2 | 2 |
| *Add something about 'how I feel about pain in the future'* |  |  | X |  |  |  | X |  | 1 | 1 |
| *Comment option for ‘time of day’ pain interferes most* |  |  | X |  |  |  |  | X | 1 | 1 |
| 1. **Make font & boxes larger with bigger spacing** | X |  |  |  | X |  |  | X | 2 | 3 |
| 1. Alternate row colours |  |  |  |  | X |  |  | X | 1 | 1 |
| 1. **Parent report for modified brief pain inventory (mBPI) but not Fear of Pain Questionnaire for Children (FOPQ-C)** | X |  |  | X |  |  | X | X | 10 | 11 |
| 1. **Response options** | X | X | X | X | X | X | X | X | 29 | 71 |
| 1. Add a colour background to the response option symbols (i.e. green to red) | X | X |  |  | X |  | X | X | 8 | 7 |
| 1. Add 'I don't know' and 'not applicable' as response options | X | X |  | X |  |  | X | X | 2 | 2 |
| 1. Add visual symbols as well as numbers |  | X |  |  | X |  | X | X | 11 | 11 |
| 1. Add words as well as numbers and visuals |  | X |  |  | X |  | X | X | 5 | 5 |
| 1. Change the number of response options to less than 10 (0-5 and/or 0-3) | X | X |  |  | X |  | X | X | 5 | 5 |
| 1. Consider changing strongly agree and disagree to a frequency scale (e.g. a lot, a little) |  |  |  |  |  | X | X |  | 1 | 1 |
| 1. Ensure it is switch accessible |  | X |  |  | X |  |  | X | 2 | 1 |
