# Supplementary material for: Examining tools for assessing the impact of chronic pain on emotional functioning in children and young people with cerebral palsy: stakeholder preference and recommendations for modification
Source: Qual Life Res. 2024 May 25;33(8):2247–59. doi: 10.1007/s11136-024-03693-1 (PMC11286630; doi:10.1007/s11136-024-03693-1)
Supplement: Supplementary file 7 — Supplementary Material 7 [file 11136_2024_3693_MOESM7_ESM.docx]

Supplementary material 3: Survey questions and their relevance to the content validity and feasibility domains:

| Question | Domain | Participant type |
| --- | --- | --- |
| 1. The [insert tool here] items are relevant and meaningful to people with cerebral palsy | Relevance | All |
| 1. The item and response option wording is clear and easy to understand | Comprehensibility | Parents/people with CP |
| 1. The time the [insert tool here] takes to complete is feasible | Clinical feasibility | All |
| 1. The way the [insert tool here] is administered (written questionnaire, interview, etc.) is appropriate and feasible | Clinical feasibility | All |
| 1. The [insert tool here] could be used with my communication device | Clinical feasibility | People with complex communication needs |
| 1. The [insert tool here] could be used with my child’s communication device | Clinical feasibility | Parents of children with complex communication needs |
| 1. The [insert tool here] requires adaptation to be appropriate for people with cerebral palsy | Need for modification | All |
| 1. I would consider using the [insert tool here] in clinical practice | Clinical feasibility | Clinicians |
| 1. I would be happy to complete the [insert tool here] as part of an assessment | Clinical feasibility | Parents/people with CP |
