# Supplementary material for: Examining tools for assessing the impact of chronic pain on emotional functioning in children and young people with cerebral palsy: stakeholder preference and recommendations for modification
Source: Qual Life Res. 2024 May 25;33(8):2247–59. doi: 10.1007/s11136-024-03693-1 (PMC11286630; doi:10.1007/s11136-024-03693-1)
Supplement: Supplementary file 8 — Supplementary Material 8 [file 11136_2024_3693_MOESM8_ESM.docx]

**ONLINE RESOURCE 1 – Decision making re: inclusion of tools in online survey**

Available pain tools measuring impact of pain on emotional functioning were identified from three recent systematic reviews [1-3] and examined using the eligibility criteria in Table 1. Pain coping tools were defined as those that assessed pain coping, including pain anxiety, pain catastrophising and fear of pain within a dedicated scale or subscale [4, 5]. Multidimensional tools were defined as assessing many pain domains in CP, with at least one item assessing impact of pain on emotional functioning, defined as “the extent to which pain hinders engagement with emotional functioning” [6]. Fifteen tools were deemed eligible for inclusion (Table 2).

Advisory group members recommended no more than eight tools be presented in the survey. Consequently, three clinician-researchers and the advisory group reviewed the feasibility of the eligible tools. This iterative process was informed by 1) COSMIN feasibility guidelines [7], 2) feasibility for use with differing communication, movement and cognitive abilities and 3) dimensions of appropriateness, practicability and accessibility [8]. Eight tools were identified for inclusion (Table 3)

TABLE 1: Inclusion and exclusion criteria for tools included in the online survey

| INCLUSION | EXCLUSION |
| --- | --- |
| Multidimensional tool with at least one item assessing the ‘impact of pain on emotional wellbeing’ | Part of an existing assessment format that is unable to have question items modified or changed |
| Assesses pain coping in a dedicated scale or subscale | Assesses pain location, pain intensity or pain frequency only |
|  | Already has content validity in cerebral palsy |
|  | A modified version of the tool for cerebral palsy and/or children is available |
|  | High level evidence of ‘insufficient’ for one or more measurement property |
|  |  |

TABLE 2: List of tools that met the initial inclusion criteria in the online survey

| **Name of tool** | **Multidimensional vs pain coping** | **Age group** |
| --- | --- | --- |
| Bath Adolescent Pain Questionnaire  (self-report & parent report) | Pain coping | Adolescents (11-18 years) |
| Chronic Pain Acceptance Questionnaire - Adolescent  (parent report, self-report, short form) | Pain coping | Adolescents |
| Fear of Pain Questionnaire for Children  (self-report, parent report, short form) | Pain coping | Children, adolescents and young adults (8-25 years) |
| Pain anxiety symptom scale | Pain coping | Children & adolescents (8-18 years) |
| Pain catastrophizing scale for Children  (parent report and self-report) | Pain coping | Children and adolescents |
| Pain coping questionnaire (parent report, self-report, short form) | Pain coping | Children and adolescents 8-18 years |
| Pain response inventory  (self report) | Pain coping | Children and adolescents (9-16 years) |
| Pain stages of change questionnaire  (self-report and parent report) | Pain coping | adolescents |
| Pain vigilance and acceptance questionnaire  (self-report) | Pain coping | Children and adolescents 8-18 years |
| Pediatric Quality of Life (PedsQL) Pediatric Pain Coping Inventory  (self-report and parent report) | Pain coping | Children and adolescents 5-17 years |
| Modified brief pain inventory (proxy report and self-report) | Multidimensional | Children, adolescents and young adults (5-34 years) |
| Pain burden inventory  (self-report and parent report) | Multidimensional | Children, adolescents and young adults (7-21 years) |
| Pain interference index  (self-report and parent report) | Multidimensional | Children, adolescents and young adults (6-25 years) |
| Pediatric Pain screening tool  (self-report) | Multidimensional | Children and adolescents (8-18 years) |
| Photograph Series of Daily Activities (PHODA) youth | Multidimensional | Adolescents and young adults (13-21 years) |

TABLE 3: Excluded tools & reasoning from the online survey

| NAME OF TOOL | EXCLUSION REASON |
| --- | --- |
| PROMIS pediatric pain interference item bank & short form (self and proxy report) | Unable to modify questions/items due to development and evaluation method (IRT) |
| Cerebral Palsy Quality of Life (CPQOL) child and teen version | Already has content validity in cerebral palsy |
| CP child | Already has content validity in cerebral palsy |
| Paediatric Pain Profile (PPP) | Does not assess impact on emotional functioning, already has content validity in cerebral palsy |
| Pediatric Quality of Life (Peds QL) CP module | Does not assess impact on emotional functioning, already has content validity in cerebral palsy |
| Colour Analog Scale (CAS) | Assesses pain location, pain intensity or pain frequency only |
| Eleven point numerical rating scale | Assesses pain location, pain intensity or pain frequency only |
| Faces pain scale revised | Assesses pain location, pain intensity or pain frequency only |
| Body diagram | Assesses pain location, pain intensity or pain frequency only |
| Visual Analog Scale (VAS) | Assesses pain location, pain intensity or pain frequency only |
| Wong Baker Faces Pain Scale revised | Assesses pain location, pain intensity or pain frequency only |
| Brief Pain Inventory | Modified version included in study |
| Child Activity Limitations Interview (CALI) 9, CALI 21 (parent and self-report) | Does not assess impact on emotional functioning |
| Child self efficacy scale | Does not assess impact on emotional functioning |
| Functional Disability Inventory | Does not assess impact on emotional functioning |
| Graded chronic pain scale & graded chronic pain scale revised | Does not assess impact on emotional functioning |
| Health Utilities Index -3 (HUI3) | Does not assess impact on emotional functioning |
| Non-communicating children’s pain checklist revised (NCCPC-R) | Does not assess impact on emotional functioning |
| Pediatric Outcomes Data Collection Instrument (PODCI) | Does not assess impact on emotional functioning |
| Pediatric Quality of Life (Peds QL) Pediatric Pain Questionnaire (PPQ) | Does not assess impact on emotional functioning |
| Psychological Inflexibility Scale | Does not assess impact on emotional functioning |

Table 3: Tools included in the online survey and their primary purpose

| **Name of tool** | **Description of the tool** | **Age group** |
| --- | --- | --- |
| Bath Adolescent Pain Questionnaire  (self-report & parent report) | A tool with seven subscales, subscale five specifically addresses worries or concerns about pain. 5-point response scale is used | Adolescents (11-18 years) |
| Chronic Pain Acceptance Questionnaire – self-report short form | 8-item questionnaire assessing two constructs - activity engagement and pain willingness. A 5-point response scale is used | Adolescents |
| Fear of Pain Questionnaire for Children – short form | 10-item questionnaire assessing two constructs – fear of pain and activity avoidance, A 5-point response scale is used | Children, adolescents and young adults (8-25 years) |
| Pain catastrophizing scale for Children – self report | 13-item questionnaire assessing pain catastrophising. A 5-point response scale is used | Children and adolescents |
| Pain vigilance and acceptance questionnaire - (self-report) | 14-item questionnaire assessing pain vigilance and pain acceptance. A 6-point response scale is used | Children and adolescents 8-18 years |
| Modified brief pain inventory (self-report) | 12-item questionnaire assessing pain interference. An 11-point response scale is used | Children, adolescents and young adults (5-34 years) |
| Pain interference index  (self-report) | 6-item questionnaire assessing pain interference. A 7-point response scale is used | Children, adolescents and young adults (6-25 years) |
| Pediatric Pain screening tool  (self-report) | 9-item questionnaire assessing pain interference, pain severity, pain location and impact of pain on emotional functioning. A binary disagree/agree response option is used for items 1-8, and a 5-point scale used for item 9 to describe how much pain has been a problem | Children and adolescents (8-18 years) |

1. Kingsnorth S, Orava T, Provvidenza C, Adler E, Ami N, Gresley-Jones T, et al. (2015). Chronic Pain Assessment Tools for Cerebral Palsy: A Systematic Review. *Pediatrics*, *136*(4), e947-60. <https://doi.org/https://doi.org/10.1542/peds.2015-0273>

2. Smith NL, Smith MG, Gibson N, Imms C, Thornton Al, Harvey AR. (2023). Pain coping tools for children and young adults with a neurodevelopmental disability: A systematic review of measurement properties. *Dev Med Child Neurol*, *65*(3), 318-28. <https://doi.org/https://doi.org/10.1111/dmcn.15410>

3. Smith MG, Farrar LC, Gibson RJ, Russo RN, Harvey AR. (2023). Chronic pain interference assessment tools for children and adults who are unable to self-report: A systematic review of psychometric properties. *Dev Med Child Neurol*, *65*(8), 1029-42. <https://doi.org/https://doi.org/10.1111/dmcn.15535>

4. Simons LE, Kaczynski KJ. (2012). The Fear Avoidance model of chronic pain: examination for pediatric application. *J Pain*, *13*(9), 827-35. <https://doi.org/https://doi.org/10.1016/j.jpain.2012.05.002>

5. Fisher E, Heathcote LC, Eccleston C, Simons LE, Palermo TM. (2018). Assessment of Pain Anxiety, Pain Catastrophizing, and Fear of Pain in Children and Adolescents With Chronic Pain: A Systematic Review and Meta-Analysis. *J Pediatr Psychol*, *43*(3), 314-25. <https://doi.org/https://doi.org/10.1093/jpepsy/jsx103>

6. Karayannis NV, Sturgeon JA, Chih-Kao M, Cooley C, Mackey SC. (2017). Pain interference and physical function demonstrate poor longitudinal association in people living with pain: a PROMIS investigation. *PAIN*, *158*(6), 1063-8. <https://doi.org/https://doi.org/10.1097/j.pain.0000000000000881>

7. Prinsen CAC, Mokkink LB, Bouter LM, Alonso J, Patrick DL, de Vet HCW, et al. (2018). COSMIN guideline for systematic reviews of patient-reported outcome measures. *Quality of Life Research*, *27*(5), 1147-57. <https://doi.org/10.1007/s11136-018-1798-3>

8. Smart A. (2006). A multi-dimensional model of clinical utility. *International journal for quality in health care : journal of the International Society for Quality in Health Care*, *18*(5), 377-82. <https://doi.org/https://doi.org/10.1093/intqhc/mzl034>
