# Supplementary material for: Examining tools for assessing the impact of chronic pain on emotional functioning in children and young people with cerebral palsy: stakeholder preference and recommendations for modification
Source: Qual Life Res. 2024 May 25;33(8):2247–59. doi: 10.1007/s11136-024-03693-1 (PMC11286630; doi:10.1007/s11136-024-03693-1)
Supplement: Supplementary file 9 — Supplementary Material 9 [file 11136_2024_3693_MOESM9_ESM.docx]

**Supplementary material 4 – Interview guide**

Before starting focus group or interview

Press record on zoom meeting

**Main themes**

*Present participants with each of the assessment tools to be reviewed, one at a time. There will be additional information about each tool (e.g. approx. time to complete, any resources required, etc.). This can be presented by sharing the screen on zoom. The participants will also have been emailed a copy of the tools prior to the focus group or interview (ideally maximum 2 tools), along with 3-5 broad questions to consider before attending*

**INFORMATION ABOUT THE ASSESSMENTS:**

*Participants will be provided with this information before the groups, and it would also be good to briefly highlight what it assesses (e.g. – highlight that the fear of pain questionnaire would be used* ***after*** *identifying that pain was impacting on daily life , to then see if pain related fear was also impacting).*

1. **Fear of Pain Questionnaire for Children**

What does it assess?

This questionnaire assesses **pain related fear** in children, adolescents and young adults with chronic pain. It covers the following pain domains:

- Impact on emotional wellbeing
- Impact of pain on physical ability
- Impact of pain on participation

How to complete it?

- Written or spoken questionnaire
- Proxy report and self-report versions available

When would it be used?

- It would most likely be used after a screening assessment had already been completed. If the screening assessment showed that pain was impacting day to day life, then it might be used to see if the **fear of pain** was also impacting day to day life.

1. **Modified Brief Pain Inventory**

What aspects of pain does it assess?

This questionnaire **screens how pain interferes with function**, including the impact of pain on emotional wellbeing. This tool has been used in children, adolescents and adults with chronic pain and disability. It covers the following pain domains:

- Impact of pain on emotional wellbeing
- Impact of pain on physical ability
- Impact of pain on participation
- Impact of pain on quality of life
- Impact of pain on sleep

How to complete it?

- Written or spoken questionnaire
- Proxy report and self-report versions are available

When would it be used?

- It would most likely be used as a ‘quick check’ for all people with cerebral palsy at medical or therapy appointments, at least yearly. It would be used to see if pain was an issue, and if so to start a conversation about how it can be better managed.

**Focus group Questions – modified Brief Pain Inventory**

1. Which questions are relevant to pain for people with cerebral palsy? (Relevance)
   - Which questions are not relevant to pain for people with cerebral palsy?
   - How could we improve the relevance of the questions?
2. How easy to understand are the questions? (Comprehensibility)
   - How could we improve the questions to make them easier to understand?
3. How relevantl are the answers?? (Relevance)
   - How could we improve the answers to make them more relevant?
4. How easy to understand are the answers? (Comprehensibility)
   - How could we improve the answers to make them easier to understand?
5. Is there anything important or unique to people with cerebral palsy that is missing from this questionnaire?(Comprehensiveness)
   - Of the suggestions you have made, which would be the most important to include?
6. What suggestions would you make to improve the layout of this tool? (Comprehensibility, clinical utility)
7. How would you feel if you were asked to complete this tool before or during an appointment with your health professional? **(individuals with CP/parents only)** (Clinical utility)
   - When would you want to complete this tool? (e.g. before an appointment, at an appointment, after an appointment)
   - How would you like to discuss the results of this questionnaire? (e.g. in the appointment with the health professional, over the phone etc.)
8. How would you incorporate a tool like this into your clinical practice? **(clinicians only)** (Clinical utility)
   - What features would need to be changed to make it feasible to use in clinical practice?
   - What current features make it useful for clinical practice?
9. What do you like about the questionnaire overall? (any area)
10. What do you not like about the questionnaire overall? (any area)
11. Out of a scale from 1 to 10, with one being of little use and 10 being extremely useful, what global rating would you give the questionnaire? (any area)

**Questions – Fear of Pain Questionnaire**

1. Which questions are relevant to pain-related fear for people with cerebral palsy? (Relevance)
   - Which questions are not relevant to pain-related fear for people with cerebral palsy?
   - How could we improve the relevance of the questions?
2. How easy to understand are the questions? (Comprehensibility)
   - How could we improve the questions to make them easier to understand?
3. How relevantl are the answers?? (Relevance)
   - How could we improve the answers to make them more relevant?
4. How easy to understand are the answers? (Comprehensibility)
   - How could we improve the answers to make them easier to understand?
5. Is there anything important or unique to people with cerebral palsy that is missing from this questionnaire?(Comprehensiveness)
   - Of the suggestions you have made, which would be the most important to include?
6. What suggestions would you make to improve the layout of this tool? (Comprehensibility, clinical utility)
7. How would you feel if you were asked to complete this tool before or during an appointment with your health professional? **(individuals with CP/parents only)** (Clinical utility)
   - When would you want to complete this tool? (e.g. before an appointment, at an appointment, after an appointment)
   - How would you like to discuss the results of this questionnaire? (e.g. in the appointment with the health professional, over the phone etc.)
8. How would you incorporate a tool like this into your clinical practice? **(clinicians only)** (Clinical utility)
   - What features would need to be changed to make it feasible to use in clinical practice?
   - What current features make it useful for clinical practice?
9. What do you like about the questionnaire overall? (any area)
10. What do you not like about the questionnaire overall? (any area)
11. Out of a scale from 1 to 10, with one being of little use and 10 being extremely useful, what global rating would you give the questionnaire? (any area)

After focus group

Send to CI list of people attended

Upload zoom recording to research folder

**Modifications for engaging children in the interview process**

1. Make contact with the parent prior to the interview (by phone or email) to discuss if there are particular strategies that would help their child to engage or feel more comfortable in the interview process
2. Build rapport first in the interview – this could involve having a general chat about something you know the child is interested in (i.e. something identified by the parent in the pre interview discussion). This could include asking what they did at school today, asking about a particular sport or hobby they are interested in
3. The wording of the questions can be changed if the child is having difficulty understanding the question. You could ask the parent to reword the interview question in a way that makes sense for the child, or this can be done by the interviewer.
4. Follow up questions can be used to explore the topic further. For comprehensibility questions, you can ask the child to interpret the question themselves (‘tell me what XXX means to you?’, ‘what do you think of when you see the response option XXXX?’
5. If the child is losing interest in the interview process, offer a short break or to discuss something of interest to the child (again using information from point 1)

**A note on the comprehensibility questions**

The authors recognise that ISPOR do not recommend the question ‘is XXXX easy to understand?’ when asking questions relating to comprehensibility [1]. The inclusion of this particular item was recommended by the advisory group, as they felt it was important to gain a general perspective on the ease of understanding of the questions and response options. In this study, this was felt to be more appropriate than what the item meant to a specific individual, as some of the participants (i.e. clinicians and parents) were providing their thoughts on whether the item was comprehensible for their child or for their patients with cerebral palsy, not specifically for themselves. We did, however, ask children to interpret the questions/response options as part of the interview (see point 4 in the ‘modifications for engaging children in the interview process’ above).

1. Patrick DL, Burke LB, Gwaltney CJ, Leidy NK, Martin ML, Molsen E, Ring L. Content Validity—Establishing and Reporting the Evidence in Newly Developed Patient-Reported Outcomes (PRO) Instruments for Medical Product Evaluation: ISPOR PRO Good Research Practices Task Force Report: Part 2—Assessing Respondent Understanding. *Value in Health*. 2011;14(8):978-88. <https://doi.org/10.1016/j.jval.2011.06.013>
